# Two-Dimensional Correlation of Quantum-Mechanical Infrared Spectra Reveals Order of Vibrational-Mode Positions and Predicts Classical RMSD Structural Descriptor

**DOI:** 10.1021/acs.jpcb.6c02116

**Published:** 2026-10-13

**Authors:** Robert J. Allsopp, Curtis W. Meuse

**Affiliations:** † Institute for Bioscience and Biotechnology Research, The University of Maryland, Rockville, Maryland 20850, United States; ‡ Biomolecular Measurement Division, 10833 National Institute of Standards and Technology, Rockville, Maryland 20850, United States

## Abstract

Quantum mechanics (QM) provides the fundamental basis for an *in silico* (computational) analogue to infrared (IR) spectroscopy. Quantum-chemical vibrational analysis, as used here with the normal-mode approximation, provides an approximate description of molecular vibrations and yields calculated vibrational transition wavenumbers and infrared intensities for the normal modes of a given molecular conformation. One challenge in quantum normal-mode analysis is identifying structural or functional group relationships between modes of two different molecular conformations. Phospholipids, which are of supreme biomedical importance because they form cell membranes, are ideal-sized targets for density functional theory (DFT) scaling in quantum vibrational analysis. Hundreds of QM spectra can be readily simulated by using DFT, enabling comparisons of modes across many lipid conformations. In addition, lipids are long enough in the *Z*-dimension (perpendicular to the membrane) that the ordering of molecular functional groups based on *Z*-coordinate centers can help describe the lipid conformation. Snapshots from triplicate bilayer systems in a vacuum were collected in a library of phospholipid quantum-mechanical models. The library illustrates how two-dimensional correlation spectroscopy (2D-COS) can be applied to assign normal modes to local regions and develop a particular order of functional group positions (order-of-positions) of a phospholipid structure. A k-nearest-neighbor (KNN) approach is applied to relate normal-mode wavenumbers and normal-mode *Z*-dimension positions to find the modes that are most similar in different lipid conformations. This mode-to-mode mapping enables predictions of the atomic position RMSD for pairs of lipids. A separate application of KNN allows for comparisons of many lipids against a fixed reference point. This enables fine-tuning of the reference-point wavenumber and *Z*-count values to correlate to a given functional group. The reference-point-based KNN reveals a second spectrum-to-structure relationship that shows that quantum normal modes correlate with the actual positions of the functional groups.

## Graphical abstract

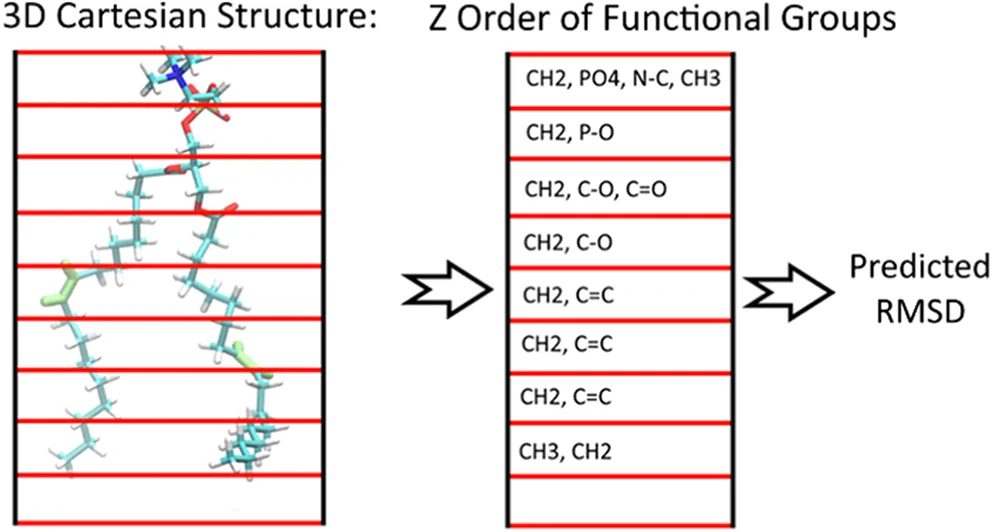

## 1 Introduction

Quantum-chemical vibrational analysis is a computational framework for predicting infrared spectra and approximates the real vibrational transitions by using the harmonic normal-mode approximation to predict the vibrational properties of a given conformation of a biomolecule. Normal modes can incorporate information about local chemical environments and differences in the order of positions that result in differences in the IR spectrum wavenumbers and absorbances.

Using phospholipid bilayers as a model system, we propose a method to expand mode annotation beyond its traditional wavenumber-matching approach by incorporating information on the ordering of functional groups along the *Z*-axis (perpendicular to the membrane). We want to be able to annotate between two QM spectra: one imagined as a hypothetical measured spectrum and another as a standard QM spectrum. To illustrate the method, we compute many infrared (IR) spectra for many conformations of a phospholipid. The IR spectra are used to predict the root-mean-square deviations (RMSD) of the structural differences of phospholipid atomic positions. This *Z*-axis information yields clearer structure–spectrum relationships, enabling comparisons of QM-derived IR spectra between conformations and comparing all spectra to reference sets of wavenumber and count. Further, this comparison framework supports the estimation of RMSD in molecular coordinate systems as quantitative measures of conformational differences.

DFT has provided critical insights into the electronic structures of atoms, molecules, and solids.^1^ The hybrid approach of Becke^2^ 3-parameter^3^ + Lee–Yang–Parr (LYP)^4^ (B3LYP) achieved sufficient accuracy to become popular in the 1990s.^1,5^ The consensus “gold standard” in quantum chemistry for single-reference QM methods is the coupled cluster singles and doubles with perturbative triples (CCSD­(T)), but calculating vibrational spectra at this level is prohibitively costly.^6,7^ The 2008 work by Krishnamurthy et al. pushed the limits of DFT to larger-sized molecules in a vacuum and included the first DFT QM spectra of any complete lipid by simulating 1,2-dimyristoyl-*sn*-glycero-3-phosphocholine (DMPC). Krishnamurthy’s calculations showed excellent agreement with the measured wavenumbers as analyzed in his report, despite using the generalized gradient approximation (GGA), and served as the starting point for this project.^8^ Although improvements in software and computing power have made the Krishnamurthy approach to simulate an entire lipid more practical for high-throughput analysis, the system size remains limited to around 200 atoms.^9^

Matching quantum mechanics (QM) and molecular dynamics (MD) simulations to experimental data is a complex process that requires fitting multiple parameters to reproduce thermodynamic properties.^10^ For example, reproducing the experimental area per lipid in bilayer models remains challenging.^11^ Even pure bilayers can be difficult to match quantitatively with experimental thermodynamic properties. Multicomponent mixtures, such as lipids and peptides, introduce additional errors into the prediction process by altering surface tension and influencing lipid conformation.^12^ MD can measure bulk characteristics such as area per lipid and localized characteristics such as changes in salt bridges, hydrogen bonds, and interaction energies to help pinpoint the locations of conformational changes.^13^ IR spectroscopy also provides sensitive signatures of molecular conformation. Since both RMSD and area per lipid provide scalar reporters of structural differences, albeit at different levels of description, it is desirable to determine how accurately QM normal modes can predict conformational differences when compared to RMSD.

QM normal-mode assignment suffers because each calculated mode is not always perfectly congruent between a measurement that captures an ensemble of conformations and a calculated conformation due to difficulty in mode-to-mode mappings without structural descriptors.^14,15^ One method for obtaining congruence in simulations utilizes Duschinsky transformations. The similarities and differences of the two conformations can be related through the Duschinsky J matrix, which describes how much mode mixing is required to transform a mode from one conformation into a mode from a different conformation. The Duschinsky transformation is an appropriate analysis for two simulated conformations, but the analysis will not be possible to perform when the comparison is between a simulation and a measurement. When comparing simulation to a hypothetical measurement, the appropriate J matrix of the measurement would not be available to perform the analysis. Therefore, Duschinsky transformations are generally beyond the scope of this paper.

Without a Duschinsky transformation, we hypothesize that mode matches between simulation and experiment can only exist if the matching process considers and accurately describes both structural and mode features across the comparison.^14^ One way to test this hypothesis for a QM-derived lipid spectrum with a measured spectrum is to compare two QM-derived spectra and make assignments based on their Cartesian coordinates (.*xyz*) weighted by a Cartesian displacement matrix. Then, the modes can be ranked from the greatest match sequentially to lesser matches. If the extent of match can be quantified and used to make predictions of the RMSD of the atomic positions of the two simulated spectra from different lipid conformations, this will support the hypothesis that incorporating structural information into the matching and analysis process is needed to compare simulation to measurement.

In measured spectra, spectral overlap is caused, in part, by the measurement of an ensemble of structures. The ensemble of structures explains some band broadening and shoulders in measured spectra where the more prevalent structures form the peaks and less prevalent structures contribute nearby; however, other contributions such as hot bands and resonances can also play important roles. In measured lipid spectra, a few well-established assignments for specific functional groups are available.^8^ Still, most assignments are predictions that require validation through extensive, time-consuming experiments.

Despite significant research, establishing cross-conformational mode correspondence remains a daunting task. One method is the mean absolute deviation (MAD cm^–1^)^14^ of normal-mode transition wavenumbers, which quantifies the discrepancy in cm^–1^ between two different spectra for a pair of peaks or compatible vibrational modes. This descriptor enables comparisons across different conformations or basis sets, as well as between simulated and measured spectra.^14^ Another approach employs thermochemistry parameters derived from the vibrational analysis, aggregating vibrational frequencies into thermodynamic quantities.^16^ In this approach, comparisons between the calculated heats of formation and measured values are used to adjust fitted model parameters and improve agreement with the measured spectra.^16^

Here, we combine a generalized 2D-COS method, initially developed by Noda,^17,18^ and a KNN approach that utilizes order-of-positions to identify corresponding modes in an automated manner.^19^ 2D-COS encompasses a family of correlation-based spectral analysis methods that are widely applied in many fields of spectroscopy,^20^ including infrared (IR),^21–24^ near-infrared (NIR),^24,25^ Raman,^23,26^ fluorescence,^27,28^ X-ray absorption,^29,30^ and nuclear magnetic resonance (NMR).^31^ The system’s spectral differences are related to changes in the molecular conformations and interactions to which the spectra are sensitive.^18,19,32^ While there is existing work comparing simulations with measured spectra,^15^ including DFT approaches that employ clustering,^8^ we present a novel approach that incorporates a new structural descriptor to augment DFT normal-mode transition wavenumbers in vibrational-mode comparisons. We explore whether a structurally based distance perturbation can enable a 2D-COS order-of-positions representation to characterize vibrational modes and their positions to reveal how interfeature distance affects the QM spectra.

While experimental lipid IR spectra assign only a limited set of functional group bands, QM calculations yield hundreds of normal modes, many of which contribute to the same experimental bands. This assignment problem is more tractable for smaller molecules with fewer modes and fewer conformations. As the molecule increases in size, it becomes more difficult to manually sort or assign all normal modes to the measured spectrum, which limits this type of analysis. Further, a bilayer system has morphological contributions that should be captured in some way. The varied geometries of lipids are shown in Figure 1, in which, lipid tails can kink. In some cases, the terminal methyl groups appear at various *z*-displacements ranging from slight overlap with the carbonyl group to penetration all the way to the core of the bilayer. This complicates the determination of the order of positions. Similarly, the choline and phosphate groups can alternate being the furthest *Z* extent of the molecule due to different conformations.

**Figure 1:**
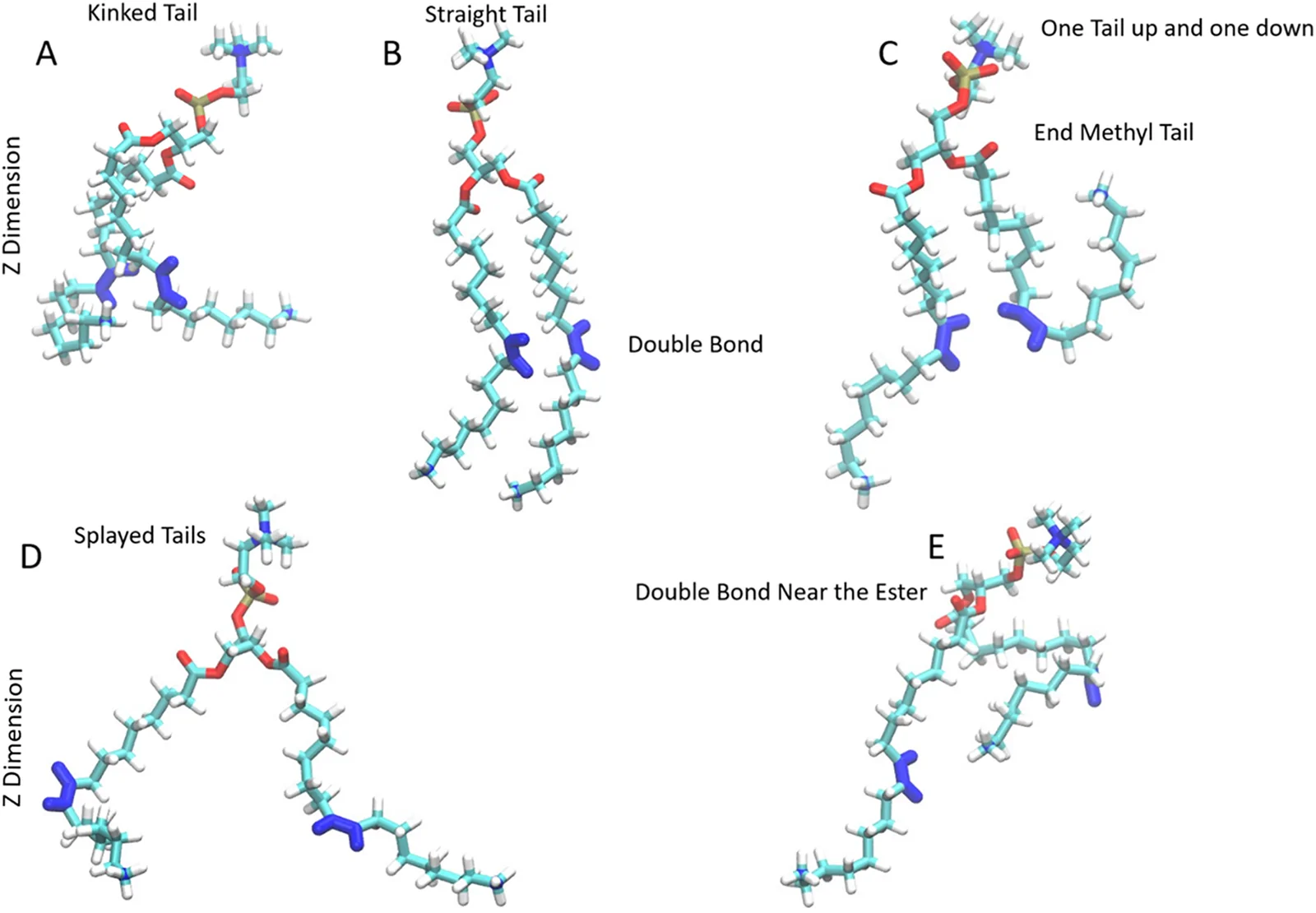
(A–E) Images of varied lipid conformations. All of these structures are from a realistic bilayer simulation. Panel (B) shows the classical extended structure of a lipid, with methyl tails positioned at the extreme along the *Z*-dimension, furthest from both the head group and the double bond, and mostly trans CH_2_ functional groups between them. In contrast, panels (A, C) show that a methyl tail can sometimes localize to the same *Z*-dimension position as the carbonyl. Panel (D) shows the lipid tails splayed far apart. Panel (E) shows that the double bond can likewise localize to the same position as the carbonyl.

Despite these challenges, we present an analysis that is general and applicable across the QM methods. The approach can compare different conformations of the same molecule and predict the RMSD. We leverage structural (positional) information in the form of *Z* and *R* (radial) order-of-positions to map the QM normal modes from one calculated conformation to another. A second variable, the QM wavenumber, is used as a secondary label to support mode matching. A k-nearest-neighbor (KNN) approach uses the Cartesian *Z* (or *Z* and *R*) coordinate order-of-positions of each vibration to apply spatial labels to the normal modes, thereby extending comparison capability between a given normal mode in one lipid conformation and similar normal modes in a different lipid molecule.

## 2 Methods^†^

### 2.1 Basic MD Setup

We create and simulate trireplicate systems of dioleoylphosphatidylcholine (DOPC) lipid bilayers. We perform full-length all-atom MD simulations of lipids using the CHARMM36 force field.^11,33^ These simulations generate the initial 3D coordinates of three replica systems built using the CHARMM-GUI *Membrane Builder*.^34–38^ The three replica bilayers had 100 lipids each. To provide ample equilibration time and to serve as the starting conformations for the calculation of quantum normal modes, we acquired snapshots of the simulations at *t* = 302 ns chosen for the ample equilibration time. Our quantum normal-mode calculations yield QM electronic structures and frequencies for all 100 lipids in each bilayer. The quickest solution to convergence issues is to run QM/MM; however, the occurrence of imaginary frequencies was low enough for DOPC to stick with the gas-phase version for this example system. The lipids in this paper were optimized in the gas phase prior to frequency calculations. However, all of the lipids with imaginary frequencies were discarded. Greater detail on the optimization type and MD settings can be found in Supporting Section 2.1.

Equilibration is demonstrated in Figure 2 by the stabilization of the area per lipid metric in the CHARMM-GUI plots for three membranes. The box dimensions were 57.8 Å × 57.8 Å × 82.6 Å. The 300 DOPC lipids were copied out of the simulated bilayers from CHARMM-GUI and processed through ORCA to derive QM normal modes of each of the individual lipids.^39–46^

**Figure 2:**
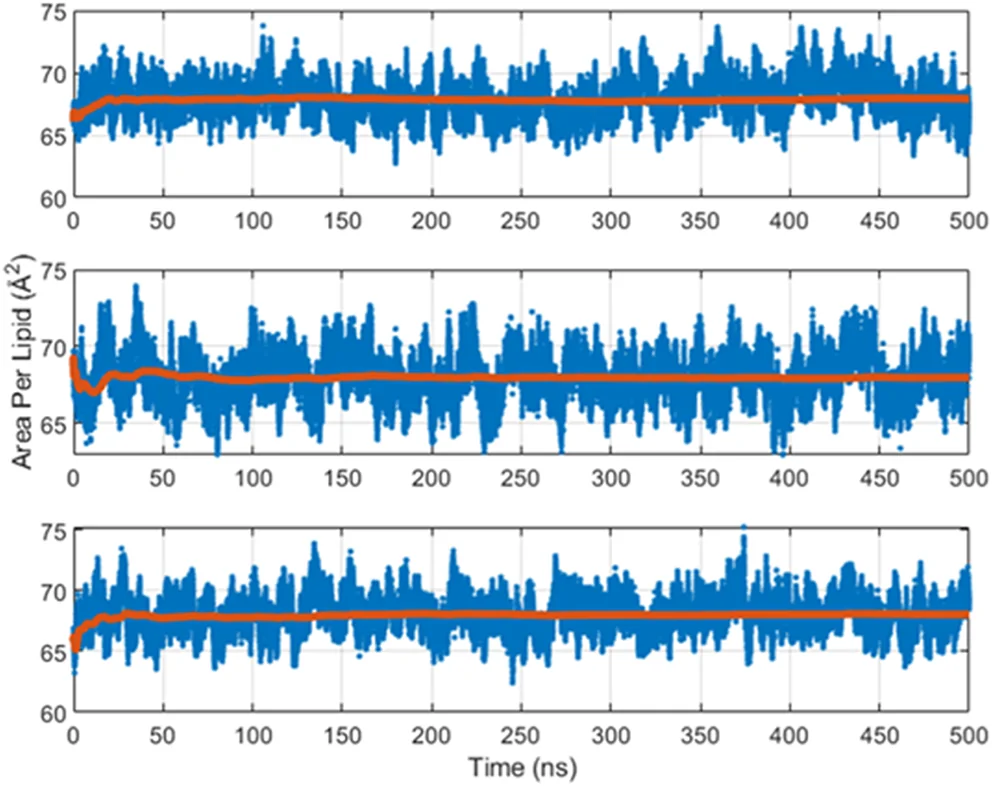
The areas per lipid of the simulated bilayers show stability and convergence across the three replicas. The order of the graphs (top to bottom) corresponds to the replica number.

### 2.2 Basic QM Setup

This section describes the system definition, the QM level of theory, the basis set and options used, and the necessary coordinate preprocessing. We ran gas-phase QM simulations of the DOPC lipid in the 2024 release of Orca 6.0.^47–57^ The work presented here generally follows the protocols of Krishnamurty, especially in the use of custom LYP correlation.^8^ Changes include the use of split-valence polarization (SVP) at the double-ζ level. Additionally, this work uses geometric counterpoise corrections.^9^ The current standards for vibrational frequency analysis range from GGA-level calculations as the highest level of theory to semiempirical frequencies at the lowest level of theory.^9^ The use of a double-ζ basis set is also acceptable, depending on the level of structural optimization.^9^ We employed the revPBE functional with default frozen-core electrons and the LYP method for correlation. This work uses the built-in Orca D4 dispersion corrections, implemented to account for van der Waals dispersion.^51–53^

The QM implementation used analytical integration to calculate frequencies and employed extreme SCF convergence, along with the “verytightopt” option, which controls several thresholds that determine the number of SCF iterations. More details on the setting and protocols and handling of exceptions like imaginary roots can be found in Supporting Section 2.2.

In Figure 3, we illustrate the system treated quantum-mechanically, a lipid with the DOPC structure. We recentered the systems using CHARMM and recentered each QM lipid one at a time before running the QM analysis and post-processing to calculate the RMSD. Recentering and Kabsch–Umeyama alignment^58–60^ are both cost-effective methods for comparing all conformations and achieving similar results, but recentering shows an improvement in *R* ^2^ predictive power of the RMSD in our work.

**Figure 3:**
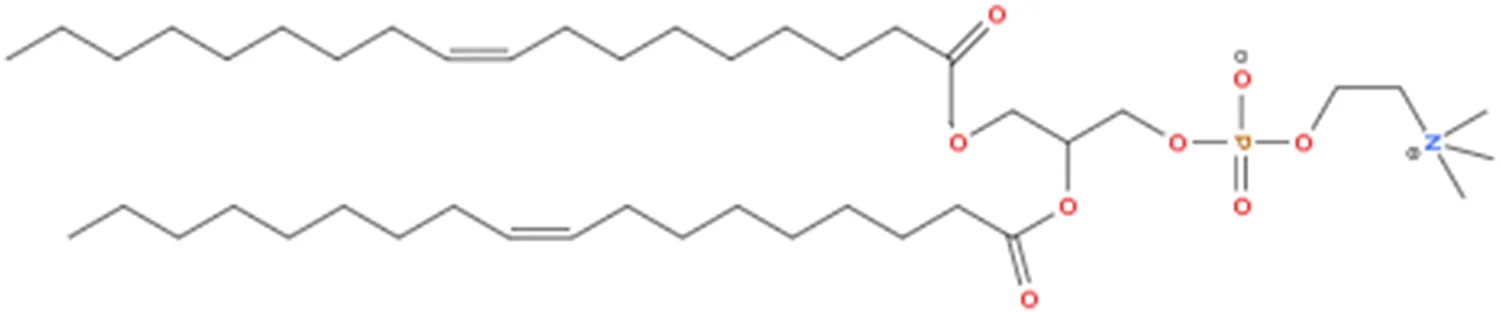
An image of the DOPC chemical structure. We generated the figure using MolView software for academic purposes.

### 2.3 QM Spectra Theory and Visualization

We calculate quantum IR normal modes from the structure and the electric field created by the molecule in question: $IR\;intensity\propto {\left(\frac{\delta \mathrm{\mu ⃗}}{\delta q}\right)}^{2}$ .^58^ Here, μ is the dipole moment and *q* is a normal coordinate that ties back to the molecules structure. A deeper discussion of the implications of this equation is presented in Supporting Section 2.3. A common practice in QM normal-mode analysis is to apply Gaussian broadening to the transition associated with a given mode to mimic the broad lines and overlap observed in measured spectra. Orca-ir from Sebastian Dechert’s GitHub was used to generate Gaussians with a fixed half-width to produce more realistic spectra for illustration purposes only; however, it should be noted that measured bands are not always Gaussian in shape.^59^

### 2.4 Assigning Distance to Normal Modes Using Orca Output

For a detailed explanation of the methods, see Supporting Section 2.4. In general, the atomic positions in the Protein Data Bank (PDB) input or the .*xyz* output (rows) need to match up with the rows in the Cartesian displacement matrix *M*(*i*,*j*). A more detailed explanation of the matrices and how they interrelate can be found in Section 2.4. The mass normalized Cartesian displacements matrix *M*(*i*,*j*) is read from the Orca output file and formatted into a pandas DataFrame matrix. The columns (index *j*) of the Orca mass normalized Cartesian displacement matrix correspond with each of the modes. $S_{NM}\left(k,j\right)=\sqrt{M{\left(k,j\right)}^{2}+M{\left(k+1,j\right)}^{2}+M{\left(k+2,j\right)}^{2}}$ , *k* = 0,3,6,...,408, which lumps the *x*, *y*, and *z* vibrational directions of each atom into one variable. The general center of mass formula ${C^{x}}_{i}=\frac{\sum_{0}^{i}y_{i}\times x_{i}}{\sum_{0}^{i}y_{i}}$ is employed to bring these matrices together. The final matrix *P*(*k*,*j*) is 138 by 3 and contains the *x*, *y*, and *z* positional information and is needed to determine the *z* and radial coordinate positions. We determine the average position of the vibration using a displacement-weighted mode center in the *z* or *r* dimension. For *z* center of mass: ${C^{Z}}_{k}=\frac{\sum_{0}^{137}{S_{NM}}_{k,j}\times P_{k,3}}{\sum_{0}^{137}{S_{NM}}_{k,j}}$ where the Hadamard product is taken for any mode *j* in the *S*_NM_(*k*,*j*) matrix. ${C^{R}}_{k}=\frac{\sum_{0}^{137}{S_{NM}}_{k,j}\times \sqrt{P_{k,1}^{2}+P_{k,2}^{2}}}{\sum_{0}^{137}{S_{NM}}_{k,j}}$ where the Hadamard product is taken for any mode *j* in the *S*_NM_(*k*,*j*) matrix. (k,1) and *P*(*k*,2) correspond to *x* and *y* information in the PDB. Where an element-by-element multiplication is taken between *S*_NM_ and the term *P*, the coordinates in matrix *P* correspond to the same atoms in the *S*_NM._ Finally, the data is normalized by dividing by the sum of the displacements for all atoms so that the displacement-normalized center for each normal mode is calculated.

### 2.5 2D-COS Order-of-Positions

The coordinate systems are defined by the Cartesian *Z*, or vertical, dimension running from the surface of the bilayer through the hydrophobic core and out to the other side. Throughout the simulation, the bilayer is free to diffuse vertically and preprocessing is required to normalize the lipid positions and orientations. Recentering is the simplest: it translates the molecule along the *x*- and *y*-coordinates, which are combined to form the radial *r*-coordinate system, and along the *z*-coordinate, as in the cylindrical coordinate system. Translations occur in space until the center of mass is at (0,0,0). After translation, the (0,0,0) coordinate usually occupies a space near the glycerol backbone. Greater detail of the preprocessing before calculating 2D-COS is included in Supporting Section 2.5.

This section describes how 2D-COS and the order-of-positions metric were computed from QM-derived vibrational spectra to enable mode-to-mode comparison across lipid conformations. We employed the 2DShige software by Morita^32^ to calculate the 2D synchronous and asynchronous 2D-COS spectra. A modified version of the recently published 2D-COS order-of-positions calculation^19^ was implemented in Python using standard pandas and numpy operations. We used two spectra to obtain the 2D-COS order-of-positions. One of the spectra is the standard wavenumber vs the intensity of the modes, in units of L/(mol·cm). We derived the second spectrum by multiplying these spectral intensities by the corresponding average QM-derived *Z* Cartesian displacement-weighted positions. The same approach was taken for the radial (*R*) dimension using the QM-derived *R* Cartesian displacement-weighted positions.

The application of positional scaling (intensity vs intensity–distance) is a novel approach in vibrational-mode analysis. We illustrate this scaling in Figure 4, where we show the orders of the modes from an origin point near the bottom or middle of the molecule, defined by the center of mass and an offset that ensures all coordinates within the lipid are nonnegative. The calculations use the mass-weighted Cartesian displacement matrix to determine how much each atom is displaced during a given vibration, thereby defining the *z* and *r* Cartesian coordinates weighted by their vibrational displacements as illustrated in the left and right panels of Figure 4, respectively. The average is taken using the normalized weights multiplied by the Cartesian *Z* or *R* component to obtain the weighted-average Cartesian *Z* or *R* component value; for example, ${C^{Z}}_{k}=\frac{\sum_{0}^{137}{SNM}_{k,j}\times P_{k,3}}{\sum_{0}^{137}{SNM}_{k,j}}$ or ${C^{R}}_{k}=\frac{\sum_{0}^{137}{SNM}_{k,j}\times \sqrt{P_{k,1}^{2}+P_{k,2}^{2}}}{\sum_{0}^{137}{SNM}_{k,j}}$ . The positions of the modes are then ordered with respect to each other in units of counts (rank index).

**Figure 4:**
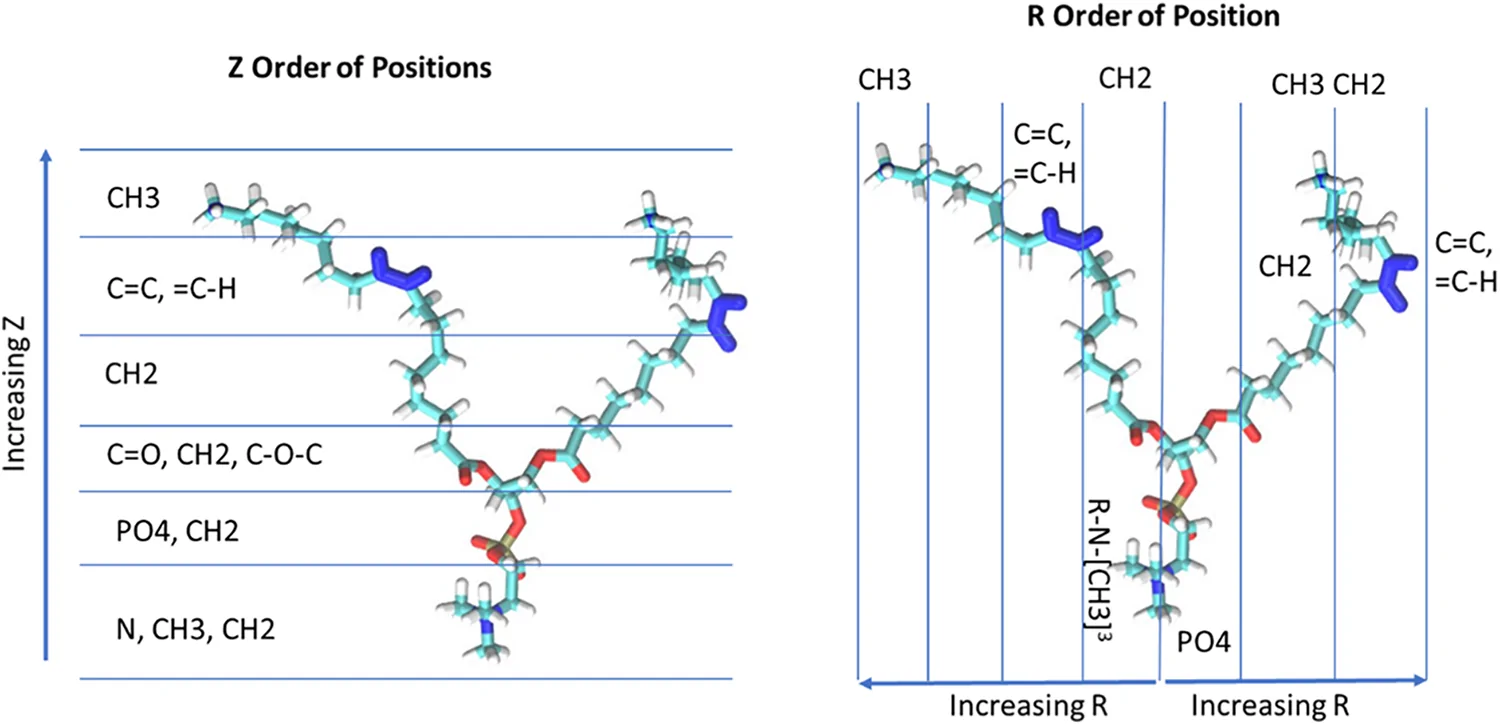
Diagram of a DOPC molecule that shows the meaning of the *Z* and *R*-coordinate systems in terms of order-of-positions and how they relate to the ordering of functional groups in the two coordinate systems.

After these scaling steps, we calculate the order of positions in units of counts. The counts are based on the number and order of the functional groups (0–407) and are limited to the last 408 possible modes. There are 414 total modes, but the first six modes correspond to translational and rotational motions. We set these translational and rotational contributions to be zero. A count is assigned to each of the 408 modes to create an index corresponding to its order positions. We base the order-of-positions on Noda’s original rules^18^ for a spectral order-of-positions, and we implement the process in an automated fashion, following established protocols.^19^

### 2.6 Use of KNN in General to Reproducibly Match Functional Groups between Simulated Lipids

We used the scikit-learn implementation of KNN to optimize the pairing of modes in one lipid conformation to the corresponding modes in another lipid. This application of KNN to mode assignment requires selecting a distance metric to measure the degree of correspondence between modes from two conformations. KNN was applied separately using the differences in wavenumber and *Z* order-of-positions values as a 2D KNN, and the difference in wavenumber and the *Z*,*R* order-of-positions as a 3D KNN. The wavenumbers and *Z*-values are never combined; but instead, their delta’s are processed in the KNN algorithm. There are two (wavenumber, Z-count) or three separate features (wavenumber, *Z* count, and *R* count). KNN effectively calculates the delta of wavenumber, *Z* count, and *R* count for all pairing possibilities and finds the pair of normal modes with the lowest delta as described in Section 3.4. No weighting of the data is needed although it can be used to prioritize the correspondence in the wavenumber to wavenumber graph or count-to-count graph. One application of KNN is to construct a matrix for each lipid pair. This KNN application is implemented algorithmically by calculating distances between all possible pairs and selecting the closest pair (*k* = 1). Once identified, the closest pair is recorded and removed from the search space. We repeat this pairing process across all modes, first for wavenumber and *Z*, and later for wavenumber, *Z*, and *R* order-of-positions. Generally, this process automatically mapped modes based on the combined differences in order-of-positions and mode wavenumbers.

### 2.7 Comparing KNN Predicted Wavenumber and Count Pairs to a Reference Functional Group *Z* Position

The centers of masses of the mode groupings of atoms, used to determine the *Z* positions of 6 test functional groups, are shown in Figure 5. In Figure 5, the dark blue sections of the lipid are the centers of mass used to define the 6 functional groups used including different CH_2_s, esters, and double bonds present on both the Sn1 and Sn2 lipid tails. This is a separate application of KNN beyond just mapping one spectrum to another and the order-of-positions that was developed earlier in this paper in Section 2.6. It is differentiated by using a fixed reference wavenumber and count for each different functional group so that all lipids are compared to the same reference points. This deeper level of comparison involved calibrating to find an acceptable count and wavenumber target that could reliably be used to correlate the positions of functional groups such as the methyl tail, choline, or double bonds between lipid conformations. More details on this approach and the target wavelength and order of positional assignments can be found in Supporting Section 2.7.

**Figure 5:**
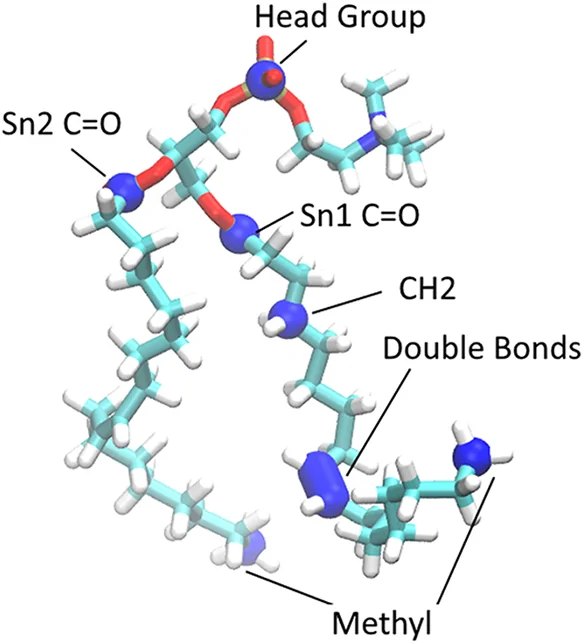
Lipid functional groups: methyl, double bond, CH_2_, carbonyls, and head group, with atoms used in the visual molecular dynamics (VMD) selection highlighted in dark blue. We consider the two esters separately because they differ in wavenumber, whereas the two methyl groups were grouped together for simplicity in this first attempt even though they can be further parsed apart.

### 2.8 Using Order-of-Positions to Predict a *Z*-RMSD and *Z,R*-RMSD

Figure 6 defines the *Z* and *R* differences (RMSD) between two conformations. We construct a filter using an *R* ^2^ cutoff of 0.99 for the *Z*-only analysis and 0.925 for the *Z*,*R*-RMSD analysis. The *R* ^2^ cutoff value for the *Z*,*R*-RMSD analysis is lower because adding the third radial order-of-positions to the KNN algorithm causes a weaker match to the *Z*-coordinate. The *Z*-coordinate is used to calculate the *R* ^2^ that determines the number of normal-mode pairs that fit on the line, also called the KNN score. We create a special case of the RMSD using recentered COM for superposition calculating only deviations in the *Z* coordinates of the atoms so that $Z‐RMSD=\sqrt{\frac{1}{N}\sum_{i=1}^{N}{\left(z_{1}-z_{2}\right)}^{2}}$ . This *Z*-RMSD was written in TCL and run in the visual molecular dynamics (VMD) software. We also considered *Z* + *R-*RMSD, which involves calculating the radial and *Z*-coordinate positions of all atoms and the differences between the two lipids. We calculated the radial values of any given atom using the formulas $r1=\sqrt{{x_{1}}^{2}+{y_{1}}^{2}}$ and $r2=\sqrt{{x_{2}}^{2}+{y_{2}}^{2}}$ and $Z,R‐RMSD=\sqrt{\frac{1}{N}\sum_{i=1}^{N}{\left(z_{1}-z_{2}\right)}^{2}+{\left(r_{1}-r_{2}\right)}^{2}}$ . A KNN score is the term used to refer to the maximum number of normal modes retained that generate an *R* ^2^ of 0.99 on the count vs the count graph. For *Z*,*R* analysis, we use three variables in the KNN: wavenumber, *Z* count, and *R* count, corresponding to the radial order-of-positions. These variables could be correlated to the MD Cartesian *Z*-RMSD and *Z*,*R*-RMSD values to reveal trends in the data. The finer details of implementing the comparison process are provided in Supporting Section 2.8.

**Figure 6:**
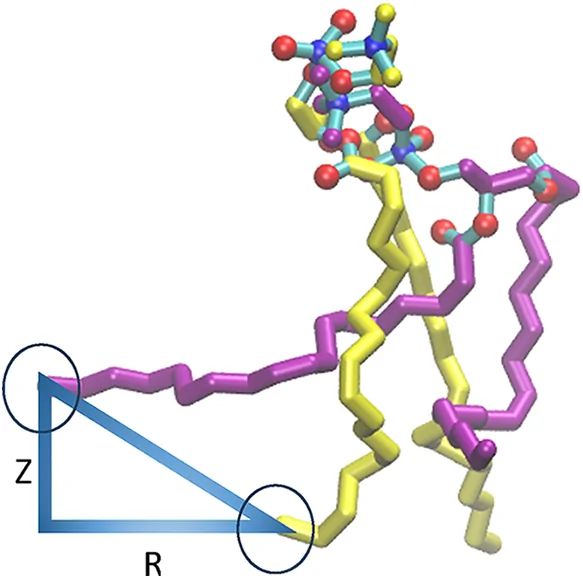
Definition of distances along the *Z*- and *R*-axes in a pair of lipids. Lipids in a bilayer are oriented in the *Z*-dimension and in the radial dimension, where $R=\sqrt{x^{2}+y^{2}}$ . Similar functional groups align along the two axes, and deviations in the *Z* and *R*-coordinate systems can quantify their differences.

### 2.9 Example GitHub Workflow

To support the development of this new tool, the complete workflow has been assembled on GitHub. The purpose is to serve as a proof of concept and to improve the accessibility of the workflow. The workflow is split into two repositories for the sake of organization. The first steps of the workflow are on the didactic pancake and feature a limited number of QM runs due to space limitations. The special-fortnight is a separate analysis. The didactic pancake includes QM input files in the directories for reference to reproduce the settings used in this study, and there could be many valid approaches to automate the QM workflow. Improvements to the workflow have been noted in the README file. The calculation of the order of positions is also included in this section for the DOPC. To change for other molecules, we need to be sure to adjust the number 414, which specifies the number of atoms times three, and all necessary adjustments needed to account for odd vs. even atom-numbered molecules. The KNN comparisons between all possible combinations of lipids are also included. Special-fortnight details how to run the single-reference KNN analysis. This analysis is paired with a standard analysis in VMD tcl that uses the measure command to append to an output file. This analysis requires uncentering the data by using the prerecorded shift applied to center the data in the first place. This is required because otherwise, the recentering will wash out any conformational differences. The didactic pancake can be found at https://github.com/rallsopp01-del/didactic-pancake, and the special-fortnight can be found at https://github.com/rallsopp01-del/special-fortnight.

## 3 Results and Discussion

Quantum normal modes can serve as descriptors of a molecule’s conformational and energy states. However, this capability remains untapped because it is challenging to compare a mode from one conformation with one from another. Our work aims to develop a method that integrates both wavenumber and spectral-order-of-position information into a process that links a mode from one set of atoms in one conformation to a similar mode in a different conformation. This linking typically uses the wavenumber to identify modes across different QM calculations and measurements. The challenge is that some normal modes’ wavenumbers match well while others do not, and there has been no practical approach to evaluate the matching process or to improve the matching of normal modes beyond fitting constants in the quantum theory.^2^ To address this limitation, we use information on the Cartesian coordinates of atoms in the form of 2D-COS order-of-positions to provide two types of information that describe any given vibration and facilitate linking two normal modes in two different conformations. For example, adding *Z*-coordinate information allows pairing vibrations with similar energy (wavenumber) while keeping the mode locations similar (*Z* order-of-positions).

### 3.1 QM Normal-Mode Calculation

Calculated normal-mode transitions have vibrational frequency and intensity. The final single-point energy of all the lipids is shown in Figure S2. For demonstration purposes, we show a plot of the normal modes for a single lipid in a single conformation from our simulations in Figure 7A. Each mode has a displacement-weighted average, located in the Z-dimension (perpendicular to the bilayer), as shown in Figure 7A. In a bilayer, lipids are distributed, with each lipid having functional groups in different conformations and localized at different distances along the *z*-axis, as illustrated in Figure 7B,C.

**Figure 7:**
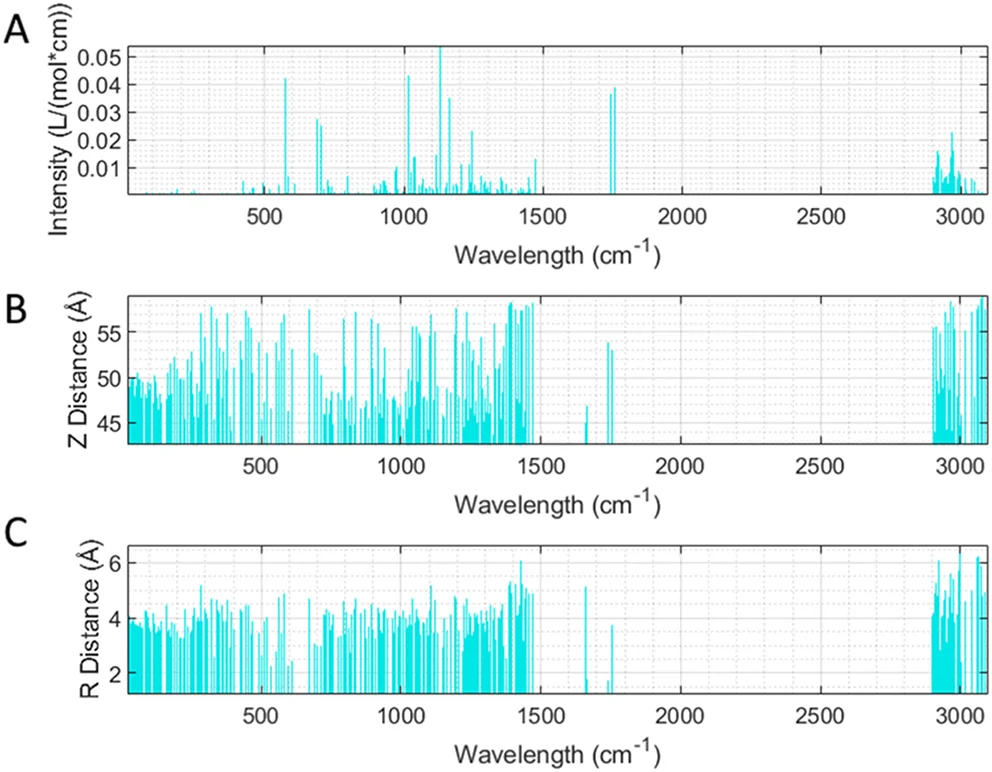
(A) IR absorption spectra of a lipid conformation. Important wavenumber assignments are similar to those documented in the literature from Krishnamurty’s 2008 work.^8^ Examples include the phosphate symmetric stretch at 1056.1 cm^–1^, the phosphate asymmetric stretch at 1226.9 cm^–1^, and the CH stretches beginning at 2868.9 cm^–1^. (B) Distance spectrum of the *Z* centers of the normal modes. (C) Distance spectrum of the mode-based *R* (radial) component centers of the normal modes.

### 3.2 Defining Order-of-Positions of QM-IR Modes

Figure 8 plots the count values from the order-of-positions vs the *Z*-coordinate position from the QM lipid .*xyz* file. These plots of order-of-positions vs the *Z*-coordinate position show monotonic behavior between the count and distance positions of the modes. This shows that the order of positions relates to distance but is not properly calibrated at each wavenumber. As shown in Figure 8, the counts of the 3*N-6 modes on the *y*-axis call out specific normal modes that can serve as probes at different Cartesian distances along the molecule. We can use these plots of the relative order of modes along the *Z*-axis to describe molecular structures. However, Figure 8 also shows that a complete description of a lipid structure requires ***a priori*** knowledge of the lipid length or the *z* positions of its modes because the density of modes is a function of distance.

**Figure 8:**
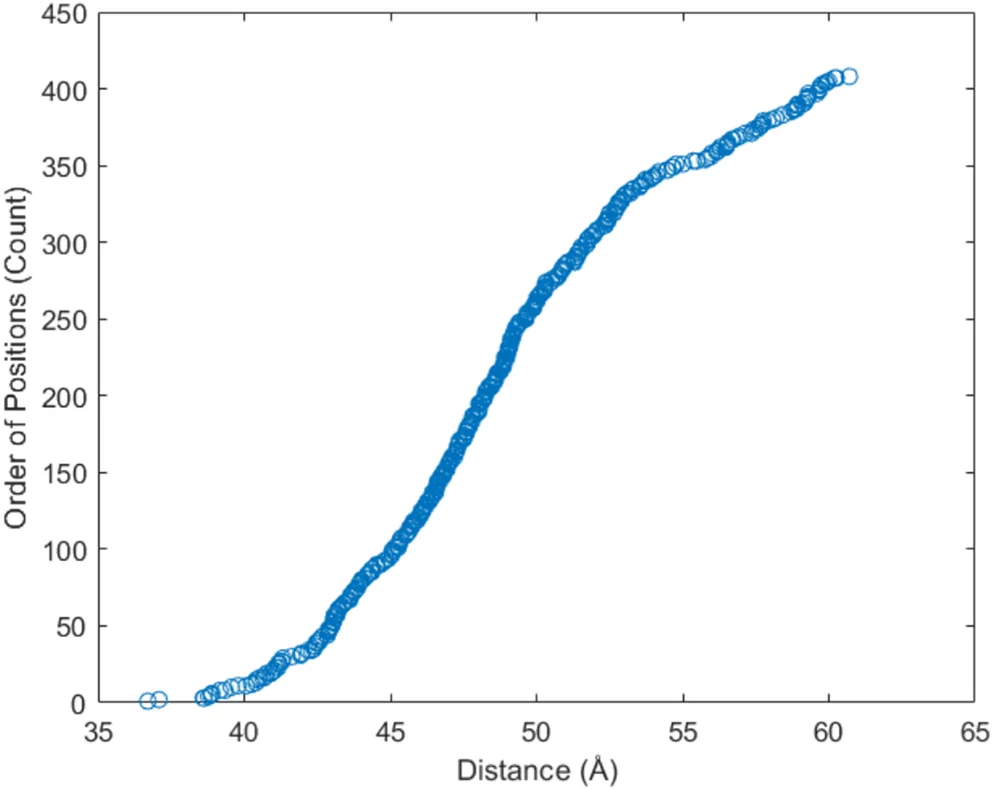
Distance vs count for a selected lipid conformation (the conformations generated by MD are numbered 1–100; shown here is data for conformation 1). The systematic increase in the order-of-positions with respect to distance shows that the order-of-positions counts encode information about the positions and distances of the normal modes in a lipid conformation. The simple sigmoidal shape of the graph suggests the possibility of establishing a quantitative calibration to calculate distance from count, but the complexity of a lipid conformation may preclude this.

It is clear from Figure 8 that the plot of distance vs the structural order-of-positions curve is not linear. Since, by definition, the counts are equally spaced, the nonlinearity arises from the unequal *Z* distance distributions of the normal modes across the lipid. For example, many normal modes have a displacement-weighted center around the middle CH_2_, above and below the double bond (48 Å–53 Å, Figure 8), with only fractions of an Å difference in their *Z* distances. On the other hand, fewer normal modes localize at the extremes of the lipid, with 2–5 Å differences between consecutive modes near 36–38 Å. This phenomenon is also discussed at the end of Section 3.3 with regard to Figure 9. These different distributions cause steeper or shallower slopes on the distance vs structural order-of-positions curve.

**Figure 9:**
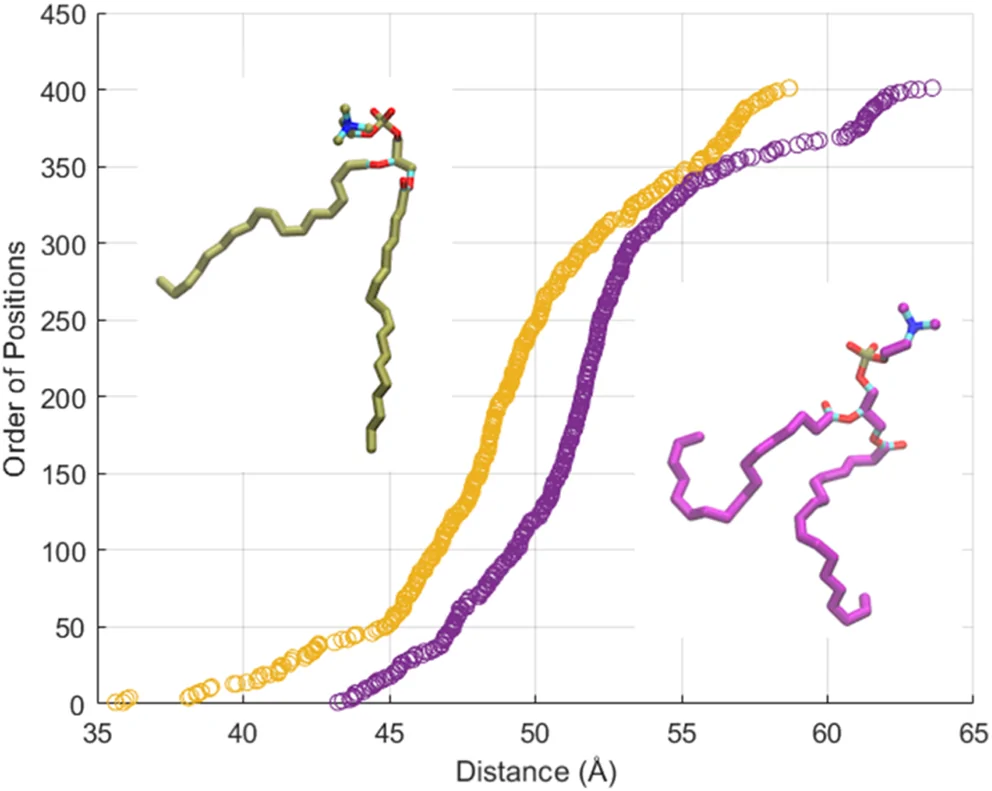
Depictions of two different lipid conformations and their order-vs-distance plots. Lipid 25 is in tan, and lipid 26 is in purple. The tan lipid corresponds to the tan graph markers that extend below 15 Å, while the purple lipid has bent lipid tails that are roughly 7 Å less extended.

### 3.3 Use of Distance vs Order-of-Positions to Compare Two Conformations

This order-of-positions vs distance plot also provides a new way to compare lipid conformations. Figure 9 demonstrates that different conformations exhibit distinct order-of-positions vs distance curves. Lipids are typically disordered and have many varied conformations of their lipid tails. Some lipid tails can have end-to-end *z* extents that are shorter, and some much longer, even within the same bilayer. The overall average lipid length is related to surface tension and lipid packing. Figure 9 shows that longer and shorter lipids with different heights yield distinctly different order-of-positions count vs distance plots. In Figure 9, we selected two lipids that show large differences in count vs distance, with the other lipids generally falling between the yellow and purple plots. For the method to work, negative *Z* coordinates are not allowed. The *Z* coordinates are shifted by subtracting the *Z* center of mass of the entire bilayer and adding an offset to ensure all Cartesian displacement-weighted *Z* positions are positive. This correction shifts the lipid up by about 10 Å, and then another 40 Å (roughly the length of the bilayer) is added on top of that. The two lipids have different heights on the distance axis and differ by about 8 Å between the upper left and lower right lipid pictures in Figure 9. The two lipids, centered on their glycerol backbones, overlap in the modes around the glycerol of the molecules. However, the two curves separate again because one choline group tilts down relative to the phosphate in the *Z*-dimension, and the other one tilts up.

Differences in the mode densities of the lipids shown in Figure 9 in the bottom left “tails” where the delta distance from one mode to another in ranked order is 1 Å–2 Å for the yellow lipid and fractions of an Å for the purple lipid. The uneven distribution of modes between the two lipids causes this. For instance, at the bottom left of the yellow plot, the fourth and fifth ordered modes are separated by 3 Å, while the modes between 45 Å and 55 Å are only fractions of an angstrom apart. Differences in the conformation of the lipid tails can also affect the density of modes and the densities of the order-of-positions. Significantly, these differences make it a challenge to know which yellow point in Figure 9 to compare with which purple point in Figure 9. A full collection of the lipids with completely successful runs is shown in Figure S3.

### 3.4 Use of KNN to Pair Normal Modes Together

Here, we demonstrate that quantum normal modes, defined solely by wavenumber and order-of-position data, contain structural information. However, recovering this structural information requires determining which mode corresponds to each order-of-positions point. Figure 10A plots the quantum normal-mode wavenumbers of lipid 26 vs the quantum normal-mode wavenumbers of lipid 25 by ascending wavenumber. Figure 10B plots the counts of lipid 26 vs the counts of lipid 25 to show the order-of-positions (structure), also paired according to the ascending wavenumber. At first glance, the comparison in the upper panel of Figure 10 shows that the quantum lipid wavenumbers for the two lipid conformations appear ordered, supporting the idea that they can be compared across different conformations. However, when a similar plot is constructed from the order-of-positions information, in the form of a count, in the lower panel of Figure 10, it becomes apparent that the wavenumbers are randomly arranged and uncorrelated with the order-of-positions. This lack of correlation means that wavenumbers alone are insufficient for assigning modes.

**Figure 10:**
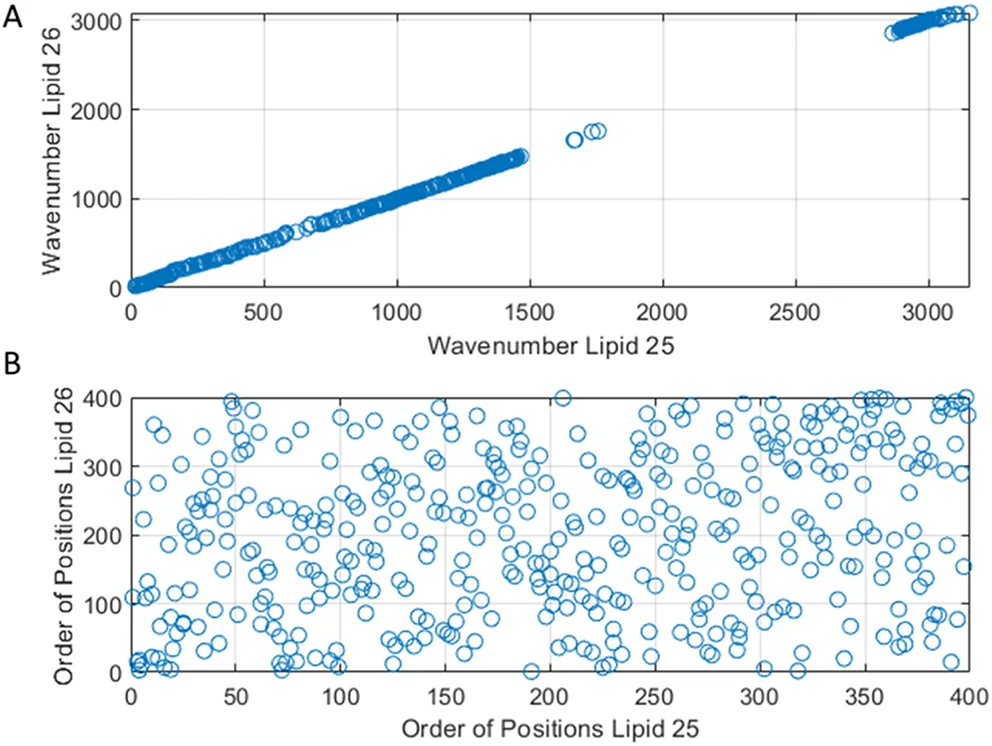
(A) Mode-to-mode comparison based on the QM wavenumbers. (B) Pairing in panel (A) is not ideal because differences in lipid conformations cause wavenumber differences large enough to completely scramble the related structural information.

In each lipid, we define each mode by both a wavenumber and a *Z* order-of-positions. Therefore, a KNN algorithm can pair wavenumbers and structural positions to identify the optimal relationships for both lipids. The KNN approach described in Section 2.5 helps identify the most similar normal mode between two conformations, based on both wavenumber and the *Z* order-of-positions.

In Figure 11, using the same plotting process as in Figure 10 after the KNN pairing, the wavenumbers and the order-of-positions are now paired in a way that minimizes the differences in both wavenumber and order-of-positions. The wavenumbers follow an approximately linear trend with deviations arising from conformational differences between the lipids that slightly shift their vibrational frequencies. The two conformations are different, so there should be differences in the absorbed vibrational energies in the wavenumber vs the wavenumber plot, and these are now visible. Further, the KNN process significantly improves the order-of-positions vs order-of-positions plot, which now shows a straight line with some off-diagonal data points. This finding is significant because the order-of-positions vs order-of-positions plot describes the conformational differences between the two lipids. After using KNN to sort the parameters, we can now compare not only the normal modes but also, for the first time, the two different conformations.

**Figure 11:**
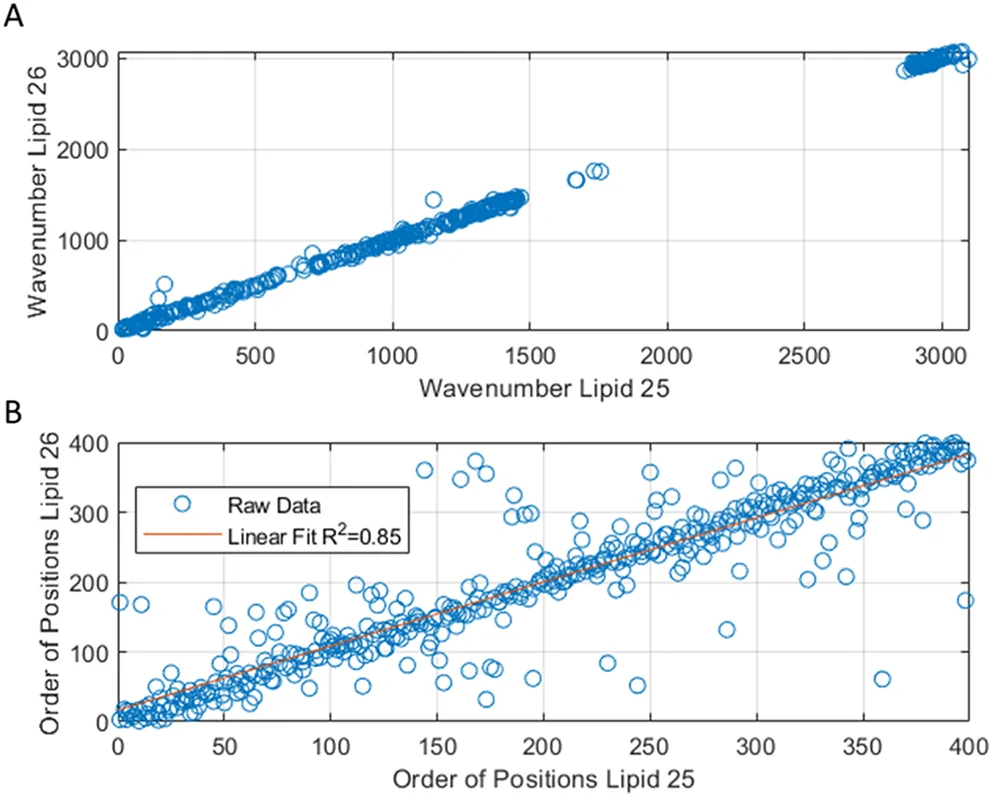
(A) Result after applying the KNN approach to compare two different lipids, showing a mostly straight line for wavenumber vs wavenumber. (B) Improvement in the linear trend of the order-of-positions count values. *R* ^2^ = 0.85 indicates a strong level of correlation.

In Figure 11, a certain number of normal modes fall within an arbitrarily small distance from a straight line, while another set of modes fall further away. We hypothesize that the number and magnitude of off-diagonal deviations predict the similarity of the two lipid structures in their *z* dimensions. The RMSD distributions of the lipids considered in this study are shown in Figures S4 and S5.

### 3.5 Use of KNN to Sort and Score Order-of-Positions Comparisons

We can also use another KNN analysis to compare pairs of wavenumbers and order-of-positions counts from each of the approximately 100 lipids in each membrane to sample different lipid conformations (84 lipids for HF-3c, and for revPBE, there were 91 lipids for replica 1, 81 lipids for replica 2, and 89 lipids for replica 3). For *N* conformations, there are *N* choose 2 or, $\frac{1}{2}n\left(n-1\right)$ , possible ways to compare the lipids or 33,930 possibilities for revPBE alone. However, this paper considers each bilayer individually, so for HF-3c, there were 3486 comparisons: 4095 for replica 1, 3240 for replica 2, and 3916 for replica 3. To support data reproducibility, the histograms of the *Z*-RMSD and *Z* + *R-*RMSD are presented in Figures S4 and S5. One benefit of working with data pairs is that the 261 revPBE QM lipids become a much larger data set. The KNN algorithm orders the data points from the best agreement with the linear trend to the worst. The desired *R* ^2^ value was achieved by removing the worst-performing pairs until the maximum set of mode pairs with an *R* ^2^ that met a threshold was calculated as defined in Section 2.7. We can then use a scoring metric that is the total number of wavenumber–count pairs retained that meet the desired *R* ^2^ value for each lipid conformation comparison to describe lipid conformation similarity.

In Figure 12, we plot the *Z*-RMSD and the number of retained KNN scores for each lipid pair to test if the quantum normal modes encode information about lipid conformation. Figure 12 also includes the same plot for *Z* and *R*-RMSD, where *Z* and *R*-RMSD are the sums of *Z*- and *R-*RMSD, rather than the *Z*-only RMSD. The plots in Figure 12 show the relationships between *Z*-RMSD and the KNN pairs that met the adjusted *R* ^2^ threshold as defined and explained in Section 2.7. If more wavenumber–count pairs exceed the cutoff threshold, then we predict greater agreement with the Z-RMSD. The plots show the expected negative correlation: lower RMSD pairs correspond to higher KNN scores.

**Figure 12:**
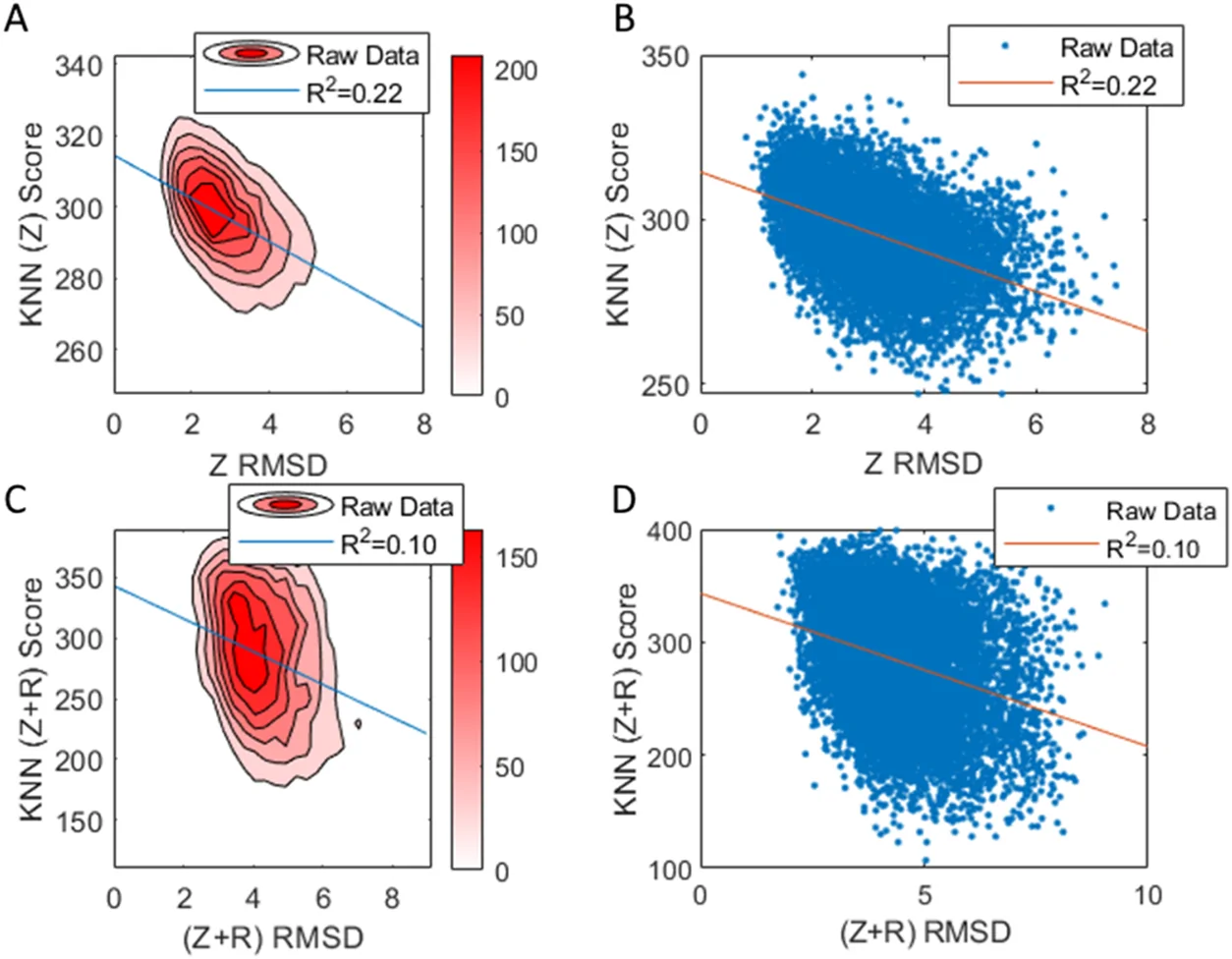
The KNN program compares all the lipid pairs to determine the number of pairs that meet the desired adjusted *R* ^2^ value of 0.99 for *Z*-RMSD and 0.925 for *Z*,*R*-RMSD. We plot the number of successful pairs against the known *Z*-RMSD values of the two lipids in the top panels (A and B) and the number of successful pairs for *Z*,*R*-RMSD in the bottom panels (C and D). The *R* ^2^ for predicting the RMSD is 0.22 for *Z* and 0.10 for *Z* *R*, suggesting that this approach is a starting point for obtaining information without knowing any of the mode positions *a priori*. The left panels (A and C) show the same data as heat maps, while the right panels (B and D) show the same data as scatter plots.

The plots in Figure 12 demonstrate how to quantify the descriptive power of these combinations of *Z*,*R* and wavenumber across lipid conformations. The overall *R* ^2^ values are 0.22 for *Z*-RMSD and 0.10 for *Z*,*R*-RMSD, indicating that a linear model explains 22% of the variance in lipid conformations. This metric assesses the KNN-derived assumption that the off-diagonal data points relate to the RMSD and the 0.99 *R* ^2^ cutoff (filter). This supports the idea that the order of positions encodes the similarity or dissimilarity of two molecular conformations. Future work could include making more assumptions to improve *R* ^2^. This approach can also test how different levels of QM theory perform in the KNN process and might justify one theory over another on the basis of an improved structural description.

### 3.6 Sensitivity Analysis of the Linear Model

Figure S6 shows the breakdown of subpopulations in the full data set, illustrating that the correlation extends across both lipid leaflets. A leaflet is the upper half or the lower half of the bilayer. Each data point is a pairwise comparison of two lipids. The *R* ^2^ values for these graphs vary from 0.13 to 0.3. The total *R* ^2^ lies in the middle between 0.13 and 0.3, at 0.22. As expected, smaller subsets of the data showed more extreme deviations from the average.

We also performed a smaller experiment to test the influence of the theory level, using the HF-3c method.^47,49,50,61,62^ The Hartree–Fock method with a three-point correction has been applied to various problems including vibrational frequency calculations. According to Figure S7, HF-3c underperforms revPBE in terms of *R* ^2^.

Pinpointing exactly what causes the different levels of theory to perform differently, with varying accuracies and fluctuations across subpopulations of the data, can be challenging but might better reveal how the analysis works and how it might compare to measurements. For now, the higher-level theory, revPBE, showed greater variation in *R* ^2^ than the HF-3c data.

### 3.7 Reproducibility of KNN Extraction of Vibrational Signatures

Finally, we tested the ability of KNN to reproducibly identify a set of matched wavenumber–count pairs of different functional groups, such as the carbonyl, head group, methyl tail, and double bonds between different lipid conformations. In Figures S8–S10, the *x*-axis shows the MD-derived Cartesian positions of the functional group, as in Figure 5. The *y*-axis shows the vibrational center of mass used to calculate counts. The wavenumber and count parts were assigned through the KNN algorithm to show how counts and KNN offer an approach to annotating the raw spectrum. There are no order-of-positions counts in Figures S8–S10.

In the correlation plots in Figures S8–S10, the hypothesis was that the actual mode *Z* positions across the collection of lipid conformations would correlate with the mode-based centers. If true, this would form a connection linking the functional groups to a specific mode or vibration and produce a straight line with a slope close to 1. The different lipid conformations would have different *Z* positions, creating a distribution, as there will always be some slight variations from other bond vibrations that cause the *R* ^2^ value to decrease. In these plots, the *R* ^2^ value indicates how consistently the target count and wavenumber match through the KNN algorithm to the correct normal-mode functional group. Figures S8–S10 show the functional groups and their linear best-fit lines for the upper leaflet of the three bilayers. Since the lower leaflet is organized in the opposite direction along the *z*-axis, plotting the two leaflets on the same graphs does not make sense. The idea is to give a sample of the data, even though an exhaustive analysis is beyond the scope of this paper. We list the *R* ^2^ of the best-fit lines in Table 1.

**Table 1:** *R* ^2^ Values for Three Different Bilayers from Figures S8–S10 ^a^

	*R* ^2^			
functional group	bilayer 1	bilayer 2	bilayer 3	mean ± standard error
carbonyl (Sn_2_)	0.75	0.82	0.73	0.77 ± 0.03
carbonyl (Sn_1_)	0.75	0.79	0.68	0.74 ± 0.03
CH2	0.56	0.78	0.65	0.66 ± 0.06
C=C	0.49	0.62	0.64	0.58 ± 0.05
CH_3_	0.73	0.67	0.78	0.73 ± 0.03
head group	0.76	0.62	0.74	0.71 ± 0.04

a The best-fit lines from the KNN pairing process compare the locations of the desired functional group across lipid conformations—the upper leaflet of the three sets of roughly 50 lipids, run at the revPBE quantum level. The standard error is the sample error divided by the square root of the number of samples.

The effort to reproducibly use KNN to extract the targeted normal mode resulted in the data found in Figures S8–S10 and Table 1, which explore the capability and its varying levels of precision for different functional groups. For example, we hypothesize that the terminal methyl group on the tail and the internal double bond will be the two most ill-matched vibrations in terms of *R* ^2^ because there are two chains and each chain can have functional groups at a range of positions. Even though they were hypothesized to be the least likely to be matched and had an *R* ^2^ value below 0.8, the matching was similar to that in the other functional group comparisons across the lipid conformations.

The expectation would have been to see an *R* ^2^ around 0.4 based on the knowledge that some methyl tails float as high as the lipid ester. This type of comparison suggests that KNN relies more on wavenumbers than on *Z* order-of-positions to identify esters and double bonds. The ester and double bonds have greater freedom and variability in their positions and corresponding count values. In those simulations, it was clear that the methyl positions vary considerably (Figure 1). These two functional groups also had the lowest mean *R* ^2^ values and the highest standard errors.

## 4 Conclusion

Lipids are important molecules that form ordered phases and exhibit lateral packing fluctuations that modulate interactions with proteins and other biomolecules. Lipid molecules are of medium size, which means that they are small enough to be computationally tractable but large enough to warrant the development of methods that address important structural questions. Despite being computationally feasible, studies of lipid structures using quantum mechanics, especially those involving calculated QM-IR normal modes, are rare. This preliminary work suggests that developing our distance–perturbation 2D-COS approach could make comparisons between QM-IR spectra and experimental IR measurements commonplace and a valuable tool for monitoring the lipid structure.

Generally, normal-mode calculations determine both wavenumbers and intensities from the vibrational transition energies. However, different conformations can have similar frequencies for different displacement patterns originating from different parts of the molecule. The approach described here applies the 2D-COS order-of-positions technique to obtain order-of-positions information from the QM-IR spectra of multiple conformations of the same lipid molecule. By adding structural information, it becomes possible to link wavenumbers and order-of-positions between two conformations of the same molecule. This 2D-COS structural information helps to improve the wavenumber comparisons of the two spectra. The correlation shown in comparisons of wavenumber to wavenumber and order-of-positions count to order-of-positions count supports the validity of our comparison process because the maximum number of KNN normal modes that form a straight line with *R* ^2^ = 0.99 is related to the *Z*-RMSD and *Z*,*R*-RMSD of the structures. The approach is valid because the number of normal modes that fall on the linear best-fit line correlates to the *Z*-RMSD of the two lipid structures.

The KNN matching of wavenumber and 2D-COS order-of-positions utilizes quantum wavenumbers as parameters to interpret lipid structures. So far, the quantum wavenumbers alone have not been used to make predictions of the RMSD of two lipids. Now, because of this work linking them to order-of-positions structural information, both order-of-positions and the wavenumber can be related to structural information. Here, the approach allows for comparison of simulated spectra. In the future, we hope that these methods serve as a starting point for applying QM methods to compare theoretical structures and spectra with measurements. Just as differences between two conformations make their modes and spectra difficult to compare, comparisons of QM modes and experimental spectra are difficult because it is impossible to know which modes are related enough to which experimental bands; including order-of-position structural information using KNN comparisons could help. Completely overcoming this challenge would directly allow first-principles vibrational calculations to describe the structural information.

## Supplementary Material



## Notes

Descriptions of the methods; three bilayers and the distribution of the number of wavenumbers above 850 cm^–1^ (Figure S1); final single point energy of the three snapshots of the bilayer and the final single point energy of the SCF optimization in Eh for reproducibility purposes (Figure S2); count vs distance spectrum for the first leaflet worth of data (Figure S3); pairwise RMSD of the different lipids from different parts of the bilayer (Figure S4); pairwise RMSD of the different lipids from different parts of the bilayer using the *Z* + *R-*RMSD (Figure S5); subsets of the revPBE data set with SVP basis set and LYP correlation—the data from the three replicate bilayer snapshots (Figure S6); subsets of the HF-3c data set with MINX basis set and D3 dispersion correction, geometric counterpoise correction, and short-range correction for deficiencies of small basis sets (Figure S7); and different functional groups selected by their wavenumber and order-of-positions of the converged lipids from 1 to 50 in bilayer 1 (Figure S8), bilayer 2 (Figure S9), and bilayer 3 (Figure S10) (PDF)
