# Supplementary material for: Two-Dimensional Correlation of Quantum-Mechanical Infrared Spectra Reveals Order of Vibrational-Mode Positions and Predicts Classical RMSD Structural Descriptor
Source: J Phys Chem B. 2026 Oct 13;130(43):11425–37. doi: 10.1021/acs.jpcb.6c02116 (PMC13726382; doi:10.1021/acs.jpcb.6c02116)
Supplement: Supplementary file 1 [file jp6c02116_si_001.pdf]

Supporting Information

Two-dimensional Correlation of Quantum Mechanical Infrared Spectra Reveals Order of Vibrational Mode Positions and Predicts Classical RMSD Structural Descriptor

Robert J. Allsopp[a,b] and Curtis W. Meuse[a,b*]

a. Institute for Bioscience and Biotechnology Research, University of Maryland

b. Biomolecular Measurement Division, National Institute of Standards and Technology, Rockville, MD 20850 USA

curtis.meuse@nist.gov

Orchid # 0000-0002-0847-6594

Keywords: quantum mechanics, density functional theory, infrared spectroscopy, generalized two-dimensional correlation

Official contribution of the National Institute of Standards and Technology; not subject to copyright

Running title: Distance Perturbations of Quantum Spectra

2.1 Basic MD Setup

The effects of the types of optimization are important considerations. Since the lipid conformations were derived from an intact bilayer the gas phase QM optimization has no steric restrictions to account for the surrounding environment so the optimization can differ from a QM/MM optimization. A QM/MM electrostatic embedding would account for the steric effects and restricting the optimization but was not used in this work. In general a gas phase optimization can find a deeper energy minimum. While the QM/MM incorporates some of the surrounding environment into the vibrational calculation to improve wavenumber and intensity assignments. We used 303.15 K for equilibration and 298 K for production runs. We employed a six-cycle equilibration protocol (50 ps to 100 ps each), based on the CHARMM-GUI output.[35-37, 39] The simulations utilized the open source NAMD software package to run the simulations for both the equilibration and the production runs. [37, 40, 41] The long-range electrostatics were treated via the particle mesh Ewald algorithm.[42] A constant-volume (NVT) ensemble for equilibration and a constant-pressure (NPT) ensemble for production runs of 500 ns, both with the Langevin piston, were used.[43-45] The simulations utilized the TIP3P water model[46] and 0.15 M KCl as the counterion. The first equilibration run used a time step of 1 fs, and the remaining runs followed the setup file on the CHARMM-GUI website. We ran production simulations with time steps of 2 fs. The restart frequency (dcdfreq), xstFreq, outputEnergies, and outputTiming were all set to 5000 steps (10 ps). The protocol used a Lennard-Jones force switching function with a 12 Å cutoff and a 10 Å switch distance. The simulations used a Langevin barostat set to 1 atmosphere, and a Langevin thermostat with a damping coefficient of 1.0 1/ps. A particle-mesh Ewald method was employed with an interpolation order of 6 and a grid spacing of 1.0 to evaluate the long-range interactions, with periodic boundary conditions.

2.2 Basic QM Setup

This resulted in the following tolerances energy change 0.0000002000, RMS gradient 0.0000080000, max gradient 0.0000300000, RMS step 0.0001000000, and max step 0.0002000000. These tolerances could be adjusted manually using the TolE, TolRMSG and TolMaxG keywords of the %geom block. Despite manually adjusting to machine precision using the tightest convergence thresholds and settings, approximately 261/300 structures returned imaginary frequencies. None of the spectra with imaginary frequencies were considered for further analysis. However, there were other issues like having an intensity so close to zero that it caused division by zero in one out of the 408 modes for ~16% of lipid samples but <1% of all modes considered. These tended to be in the frequencies below 300 cm^{-1} and the KNN moved them to the bottom of the list. These could be considered more in the future but with virtually no intensity then it is not clear that they should not be counted and that is what the KNN does.

A total of 261 unique lipids had complete non-imaginary spectra out of 300 total attempted conformations. The vibrational spectra were all run using the same conditions in the methods section. While experimentation with the parameters took place with another batch of files that are not included here. This experimentation with parameters did not cause any more conformations to converge and remove all imaginary frequencies. We attempted to improve the number of lipid structures that converged by using unpruned grids and finer grid spacing, increasing IntAcc, and increasing the cutoff tolerances to machine precision, but with no success. The first bilayer had 9 lipids that did not converge, the second had 19, and the third had 11. No imaginary modes were allowed in any of the post-processed data, as this is beyond the scope of the study. These structures that yielded imaginary frequencies were excluded to ensure the quality of the dataset.

2.3 QM Spectra Theory and Visualization

We calculate quantum IR normal modes from the structure and the electric field created by the molecule in question: $IR\ Intensity \propto \left(\frac{\delta\vec{\mu}}{\delta q}\right)^2$. [58] Where μ is the dipole moment and, q is a normal coordinate. Since the intensity is sensitive to the structure of the molecule, if two different lipid conformations were compared to each other they would yield an RMSD and two spectra that differ in wavenumber and order-of-position as defined in section 2.5. This is where annotating and relating one mode to another becomes important. Due to the quantum calculation's sensitivity to structure, it is possible that the quantum wavenumbers and intensities could predict the molecular structures' RMSDs.

A common practice in QM normal mode analysis is to apply Gaussian broadening to the transition associated with a given mode to mimic the broad lines and overlap observed in measured spectra. Orca-ir from Sebastian Dechert's GitHub was used to generate Gaussians with a fixed half-width to produce more realistic spectra for illustration purposes only; however, it should be noted that measured bands are not always Gaussian in shape.[59] Alternative visualizations show the modes as individual, unbroadened impulses, displayed either by intensity (parameter Int, km/mol) or molar absorbance (parameter EPS, L/(mol·cm)).

2.4 Assigning Distance to Normal Modes Using Orca Output

The major expectations for applying distance assignments are 1) That the Orca matrix output is that of a QM vacuum normal mode approximation 2) That the molecule has been recentered and has its center of mass set to (0,0,0). 3) That there are no negative values in Z coordinate positions due to having an offset in the Z direction when the molecule is recentered. When a unique offset is calculated after each lipid is centered by subtracting the z center of mass of the entire bilayer which sets the bilayer back to the center of the box and then adds a constant 40 Å, the length of the bilayer. 4) That the radial component also has no negative values. The atomic positions in the Protein Data Bank (PDB) input or the .xyz output (rows) need to match up with the rows in the Cartesian displacement matrix M(i,j). The PDB matrix is an input to ORCA while the M(i,j) is an output from ORCA and they differs because the PDB contains coordinates of atomic positions and M(i,j) contains the displacements from the normal modes. The PDB places the x, y, and z information of each atom in a row of the matrix so that there is a x column, a y column, and a z column. The Cartesian displacement matrix M(i,j) places each displacement in its own row, so there is a x row, y row, and z row for atom 1 followed by the x, y, and z row for atom 2. The rows of M(i,j) define the x, y, and z vibrational displacement information in groups of three to match the order of the PDB or .xyz file. Deeper inspection of the M(i,j) matrix can identify the modes with the biggest displacements by scanning the rows that correspond to the x, y, and z vibrational displacements of all atoms.

The mass normalized Cartesian displacements matrix M(i,j) exists to read in the raw displacement information from the Orca data file and is used to weight the Cartesian coordinates to determine a mode normalized center of mass. It is read in from the Orca output file and formatted into a pandas DataFrame matrix. The columns (index j) of the Orca mass normalized Cartesian displacement matrix correspond with each of the modes.

Another matrix S_{NM}(k,j), where NM stands for normal mode, is defined to process the displacement information into a directly useable matrix that is compatible with the PDB or .xyz coordinate file. The S_{NM}(k,j) uses the k index for the rows because the matrix size changes when the x, y, and z displacements are lumped together into one element for each atom when converting M(i,j) into S_{NM}(k,j) and to match the index in the P matrix. This is done by regrouping the x, y, and z elements of the M(i,j) matrix of each atom into different rows. Groups of three are formed for any given column: $S_{NM}(k,j) = \sqrt{M(k,j)^2 + M(k+1,j)^2 + M(k+2,j)^2}$ k=0,3,6…408 which lumps the x, y, and z vibrational directions of each atom into one variable. The matrix S_{NM}(k,j) is 138 (number of atoms in the system) by 3N-6=408 (number of modes in the system). The general center of mass formula $C^{x}{}_{i} = \frac{\sum_0^i y_i * x_i}{\sum_0^i y_i}$ is employed to bring these matrices together. There are 3N-6 or 408 total modes in the system. This is verified because the first six columns of M(i,j) are completely zeroed out due to rotations and translations that have been projected out of the Hessian before calculation.

The final matrix P(k,j) is 138 by 3 and contains the x, y, and z positional information and is needed to determine the z and radial coordinate positions. The three x, y, and z coordinates correspond to the three columns of j. Additionally, the values of k in P(k,j) correspond with the atoms in the matrix and match up with the kth atom in matrix S_{NM}(k,j).

We determine the average position of the vibration using a displacement-weighted mode center in the z or r dimensional space. For z center of mass: $C^{Z}{}_{k} = \frac{\sum_0^{137} S_{NMk,j} * P_{k,3}}{\sum_0^{137} S_{NMk,j}}$ where the Hadamard product is taken for any mode j in the S_{NM}(k,j) matrix. The elements from $S_{NMk,j}$ and $P_{k,3}$ are multiplied together to form a vector which is summed together for each mode (j) and divided by the sum of $S_{NMk,j}$. P(k,3) corresponds to the third column of coordinate information in the PDB file. The third column contains the Z information in the PDB matrix P(k,j).

For R center of mass: $C^R{}_k = \frac{\sum_0^{137} S_{NMk,j} * \sqrt{P_{k,1}^2 + P_{k,2}^2}}{\sum_0^{137} S_{NMk,j}}$ where the Hadamard product is taken for any mode j in the S_{NM}(k,j) matrix. k,1) and P(k,2) corresponds to x and y information in the PDB. Where an element-by-element multiplication is taken between S_{NM} and the term P so that the coordinates in matrix P correspond to the same atoms in the $S_{NM.}$ Finally, the data is normalized by dividing by the sum of the displacements for all atoms so that the displacement normalized center for each normal mode is calculated.

2.5 2D COS Order-of-Positions

Later, when calculating the order-of-positions, a Z offset is applied that shifts all Z coordinates to positive values, and the center of mass of the entire lipid is no longer equal to the origin. This approach has the advantage of not requiring assumptions about the orientation of the lipid beyond the center-of-mass step, and it assumes that all lipids have their head groups at the top and their tails at the bottom, or vice versa. The lipids are flipped to enforce this effect for consistency. Another possibility is to align the molecules. Alignment[60] is more complex and involves minimizing distances along the x, y, and z dimensions, while also rotating and reorienting the lipid. However, this requires making assumptions about rotation of the molecule.

The Z and R order-of-positions are calculated using 2D correlation methods and rank the vertical and radial-dimensional orders of vibrational modes, as grouped by quantum normal modes. We calculate the extent of vibration by normalizing the weights to 1, multiplying the normalized weights by the Cartesian Z or R coordinate. In the last step, we retrieve the Cartesian Z and R coordinates from the .xyz file and combine them with the displacement matrix using the weighted average equation.

2.7 Comparing KNN Predicted Wavenumber and Count Pairs to A Reference Functional Group Z Positions

The first step in the correlations between all lipids in a leaflet involved running the KNN to search for the closest matching pair in the data set. This process compares any given lipid to an imaginary functional group that is held fixed in space with artificial target wavenumber and count values from a predefined list. The values in the list were calibrated to correlate with the functional groups of all the lipids instead of just matching pairs of lipids where every pair is different. Without passing the data through any computer algorithm and using manual optimization it was possible to find adequate values for the count and wavenumber values. The next step involved cross referencing to find the Z position according to the Cartesian values weighted by the displacement matrix associated with that count and wavenumber pair. Finally, the mode-based distance and the atomic based distance were compared with each other.

Specifically, the wavenumber and count values that were used in our analysis to correlate with the functional groups include: methyl (3001.47 cm^{-1}, 408 count), double bond (975.64 cm^{-1}, 176 count), CH_2 (1301.02 cm^{-1}, 127 count), Sn_1 C=O (1735 cm^{-1}, 77 count), Sn_2 C=O (1703 cm^{-1}, 77 count), and head group (1039.61 cm^{-1}, 72 count) The lowest count comes last, and the highest count comes first when working the way up from the bottom as illustrated in Figure 4. . KNN is used to cross reference a given wavenumber and count coordinate between all the lipid structures. The hypothesis is that KNN can reproducibly identify functional groups by their wavenumber and count. To evaluate how well they match, a known position of each functional group in one lipid is used to form a correlation plot. The numbers presented here are modified for consistency between the lipid conformations.

The first modification is that wavenumbers below 850 cm^{-1} were excluded so the total number was not actually 408 but instead a number around 265. The number changed from trial to trial because the

number of modes above 850 cm^{-1} varied some as shown in Figure S1. Secondly there is some mismatch in the size of count and wavenumber space. The wavenumbers range from 0 to 3100 and the counts range from 0 to 400. Without reweighting the data in some way the KNN would be much more sensitive to wavenumber than to counts. The results were scaled so that the highest count number was 2500 which affects the weighting or how much preference is given to matching the counts vs the wavenumbers. Multiplying by 2500 shifts focus a bit towards the order-of-positions (counts) differences although they were not perfectly normalized because counts are evenly distributed and wavenumber has a gap gap between approximately, 1800 cm^{-1} and 2800 cm^{-1} where no modes have wavenumbers.. This was chosen on purpose because in some cases, like the methyl tail, there is a lot of variability and it is not advantageous to be too locked into any given count and likely better to consider the wavenumber more. The numbers presented here were converted back by using the ratio 408/2500 to keep with the idea of having 408 normal modes in total.

2.8 Using Order-of-Positions to Predict a Z-RMSD and Z, R-RMSD

Different conformations yield different order-of-positions and wavenumbers, that can be correlated to yield differences in RMSD. Molecules are typically aligned during RMSD calculations, so it could make sense that KNN is the counterpart to comparing vibrations with each other. A challenge in working with QM normal modes is linking a mode from a mode with certain cartesian displacements of atoms in one lipid to a similar mode in another lipid with a different conformation. This problem becomes even more unwieldy when one tries to link all possible modes simultaneously between two different molecules. KNN was used to optimize the pairing of wavenumbers and counts between different lipid pairs in a Python script. These pairs of "order-of-positions of lipid 1" vs "order-of-positions of lipid 2" could then be plotted to describe the conformational differences between the two lipids. These order-of-positions vs order-of-positions plots were then fit to a linear line to quantify the similarity of the two lipid conformations.

We create comparisons across the upper leaflet by a nested "For" loop that iterates over lipids 1 to 50, and the lower leaflet iterates over lipids 51 to 100. Additionally, there is a third comparison for lipids between leaflets 1 to 50 and 51 to 100, which creates more comparisons than the first two, limited to within each leaflet. We organize our lists of wavenumber-count pairs so that the best-fitting pairs (KNN distance) appear at the top, and the lower pairs show progressively worse agreement. Note that count refers to the order-of-positions of the functional groups, ranging from 0 to the number of normal modes considered. This analysis enabled the number of pairs to be adjusted. Essentially, the R^2 can approach 1 by searching for the maximum set of pairs of wavenumbers and counts starting with the best matching (KNN distance) adding as many of the lesser matching pairs until the R2 ≥ 0.99. In this exercise the ordering of the list and which part (top, middle, bottom) is accessed become crucial.

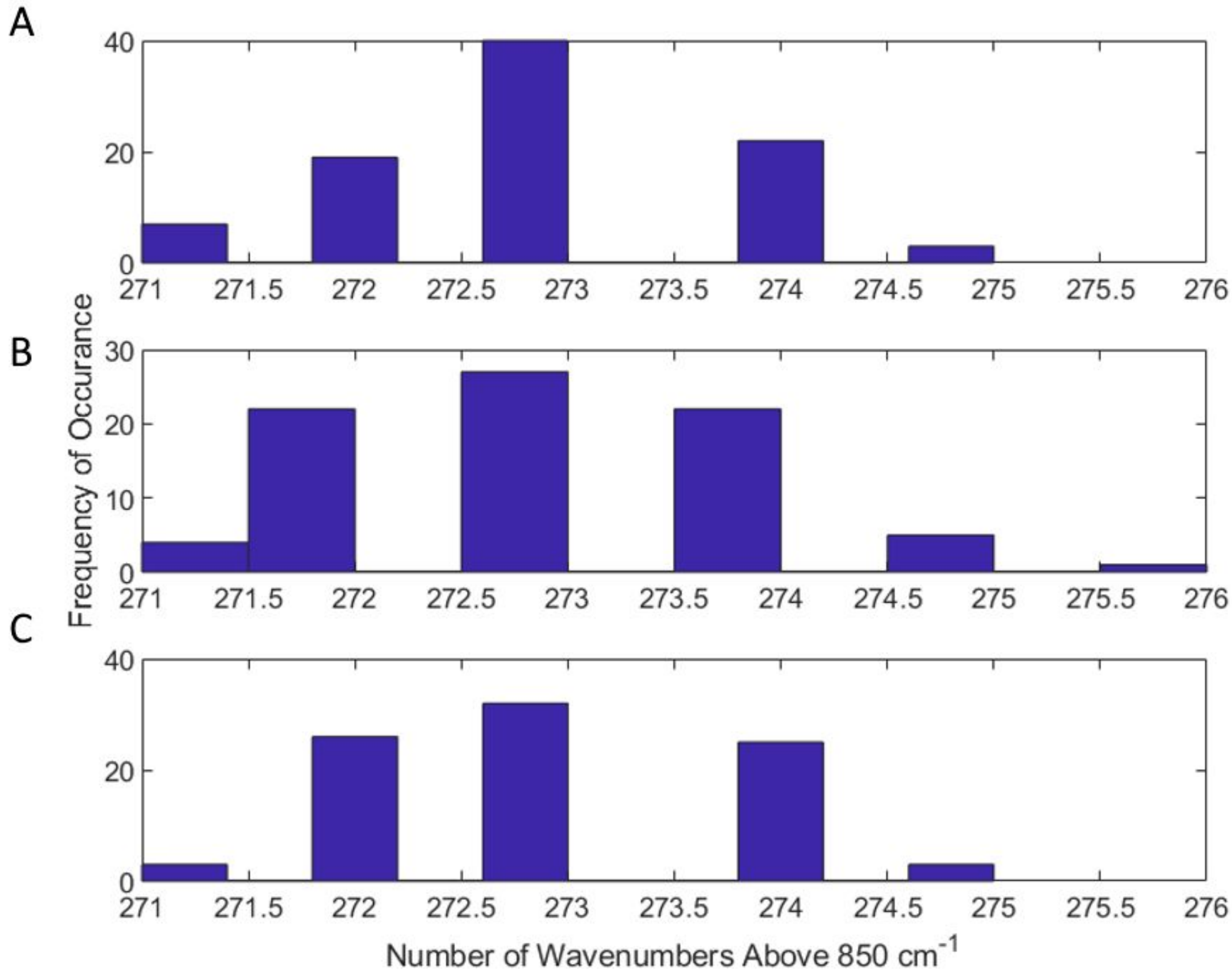

Figure S1) Shows the three bilayers and the distribution of the number of wavenumbers above 850 cm^{-1}. Panel A is membrane replicate 1, panel B is membrane replicate 2, and panel C is membrane replicate 3.

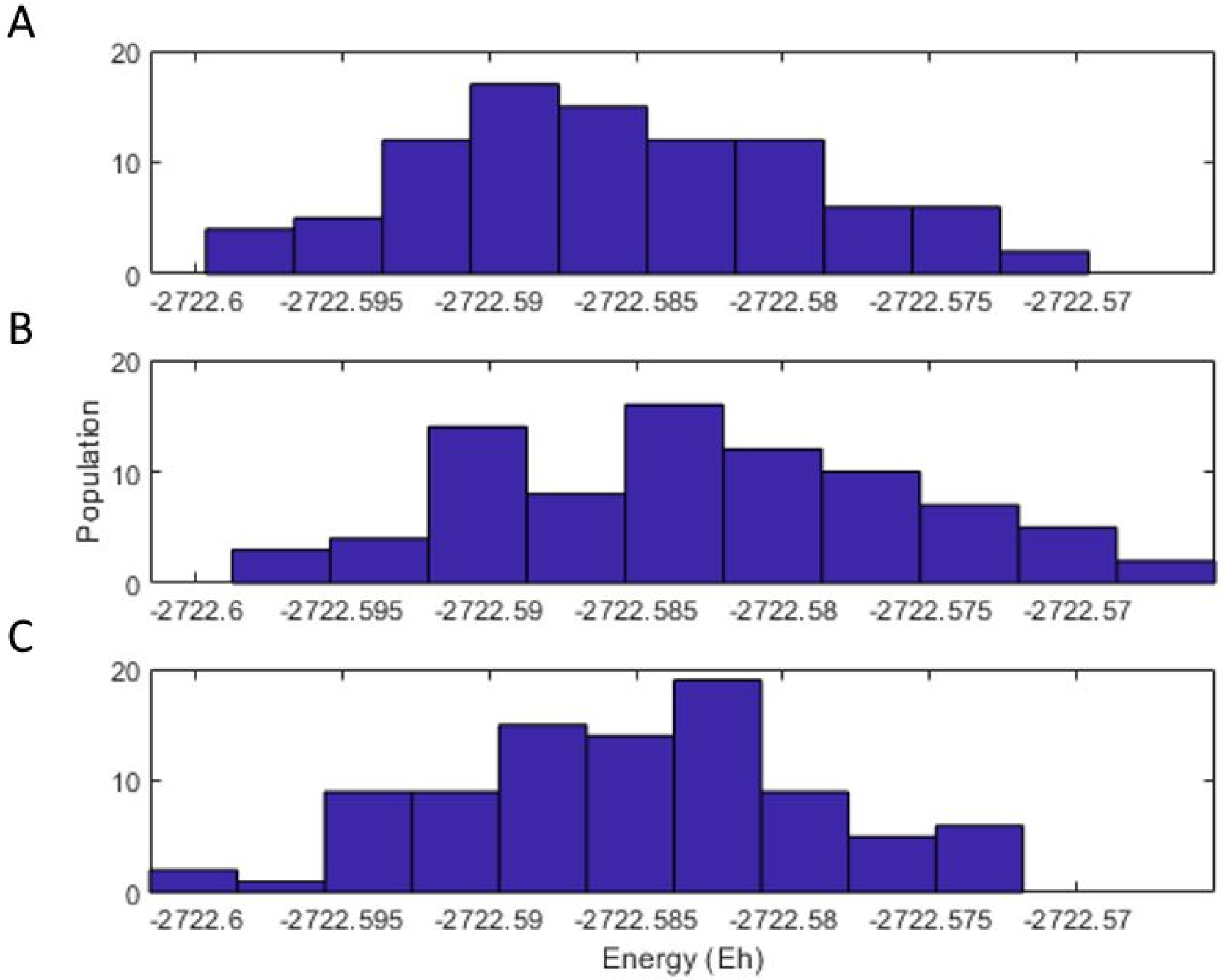

Figure S2) Shows the final single point energy of the three snapshots of the bilayer and the final single point energy of the SCF optimization in Eh for reproducibility purposes. Each histogram contains a different number of lipids. Snapshot 1 contains 91 lipids, snapshot 2 contains 81 lipids, and snapshot 3 contains 89 lipids. The different panels correspond to the different membrane replicates. Panel A shows replicate 1, panel B shows replicate 2, and panel C shows replicate 3.

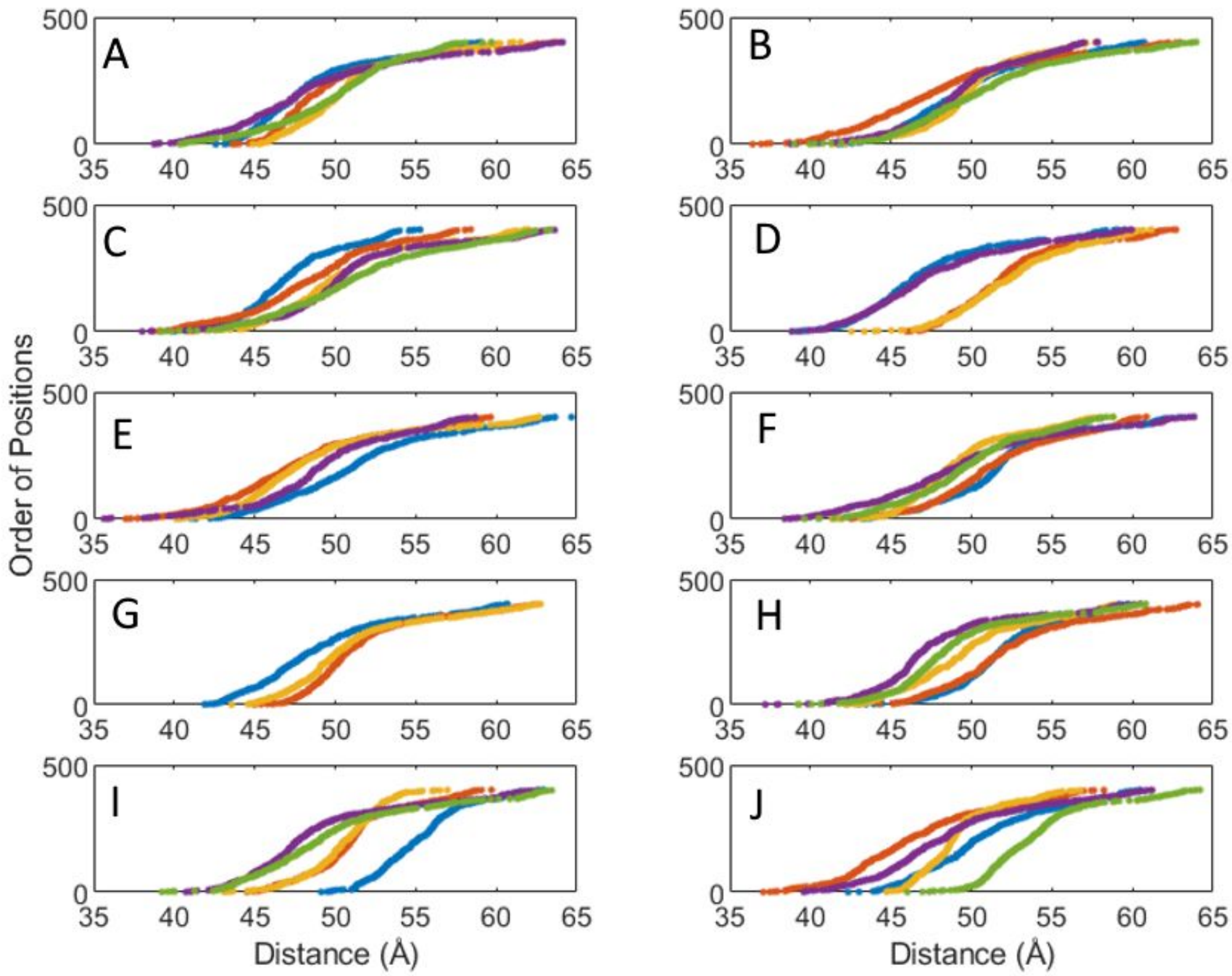

Figure S3) Shows the count vs distance spectrum for the first leaflet worth of data. This gives an idea of the structural differences from lipid to lipid. A crucial step in this analysis is uncentering the bilayer by subtracting the center of mass of the entire lipid mass so that any local lipid information is retained in the distance axis. There a total of 46 lipids included in these plots even though 10 plots of 5 spectra were intended. Eight samples with a normal mode intensity of zero that caused errors and were still included. The only four exclusions come from having imaginary modes. It is noted that even though a meaningful number of samples had modes with zero intensity the KNN still performed well. The different panels correspond to the different lipid spectra so that the first five lipid conformations are shown in panel A and the second five are shown in panel B.

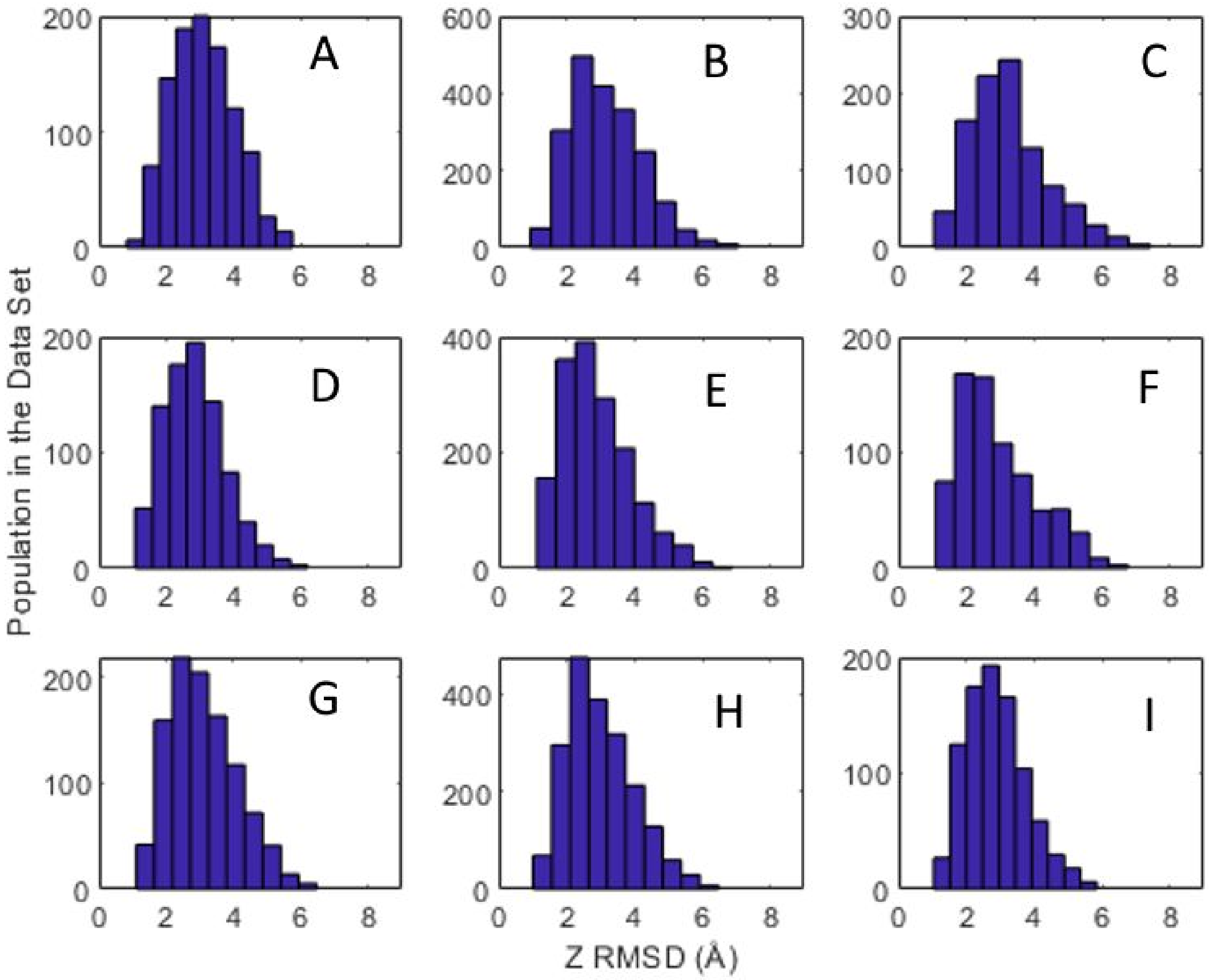

Figure S4) Shows the pairwise RMSD of the different lipids from different parts of the bilayer. The first row (panels A,B, and C) show snapshot 1, the second row (panels D, E, and F) show snapshot 2, and the third row (panels G,H, and I) show snapshot 3. The lipids are organized so that the first leaflet has lipids 1 to 50 (panels A, D, and G) and the second leaflet has lipids 51 to 100. The first column (panels A,D and G) have lipid 1 to 50 compared with 1 to 50, the second column (panels B, E, and H) have 1 to 50 compared with 51 to 100, and the third column (panels C, F, and I) have lipids 51 to 100 compared with 51 to 100.

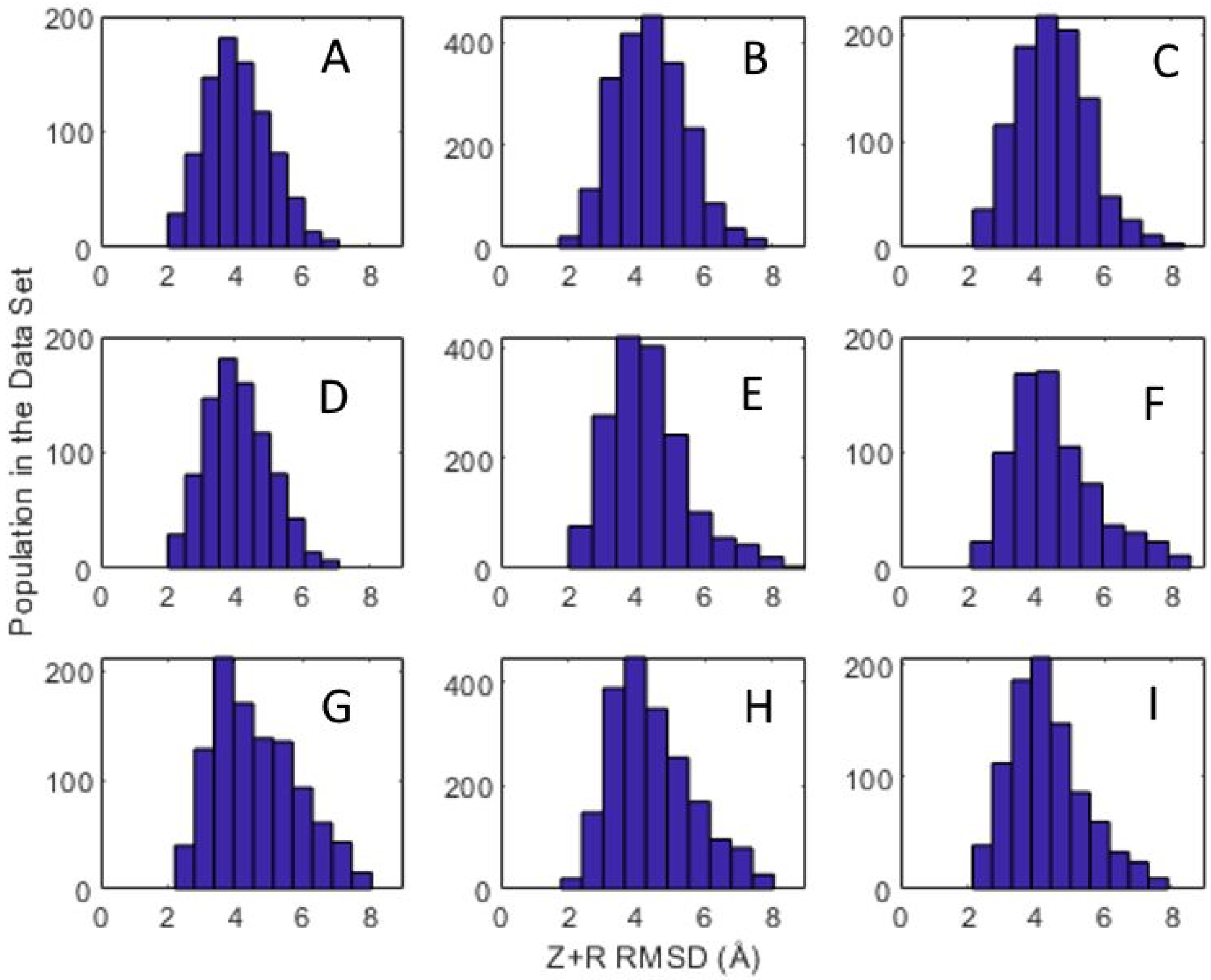

Figure S5) Shows the pairwise RMSD of the different lipids from different parts of the bilayer using the Z+R RMSD. The first row has snapshot 1, the second row has snapshot 2, and the third row has snapshot 3. The lipids are organized so that the first leaflet has lipids 1 to 50 and the second leaflet has lipids 51 to 100. The first column (panels A, D, and G) have lipid 1 to 50 compared with 1 to 50, the second column (panels B, E, and H) have 1 to 50 compared with 51 to 100, and the third column (panels C, F, and I) have lipids 51 to 100 compared with 51 to 100.

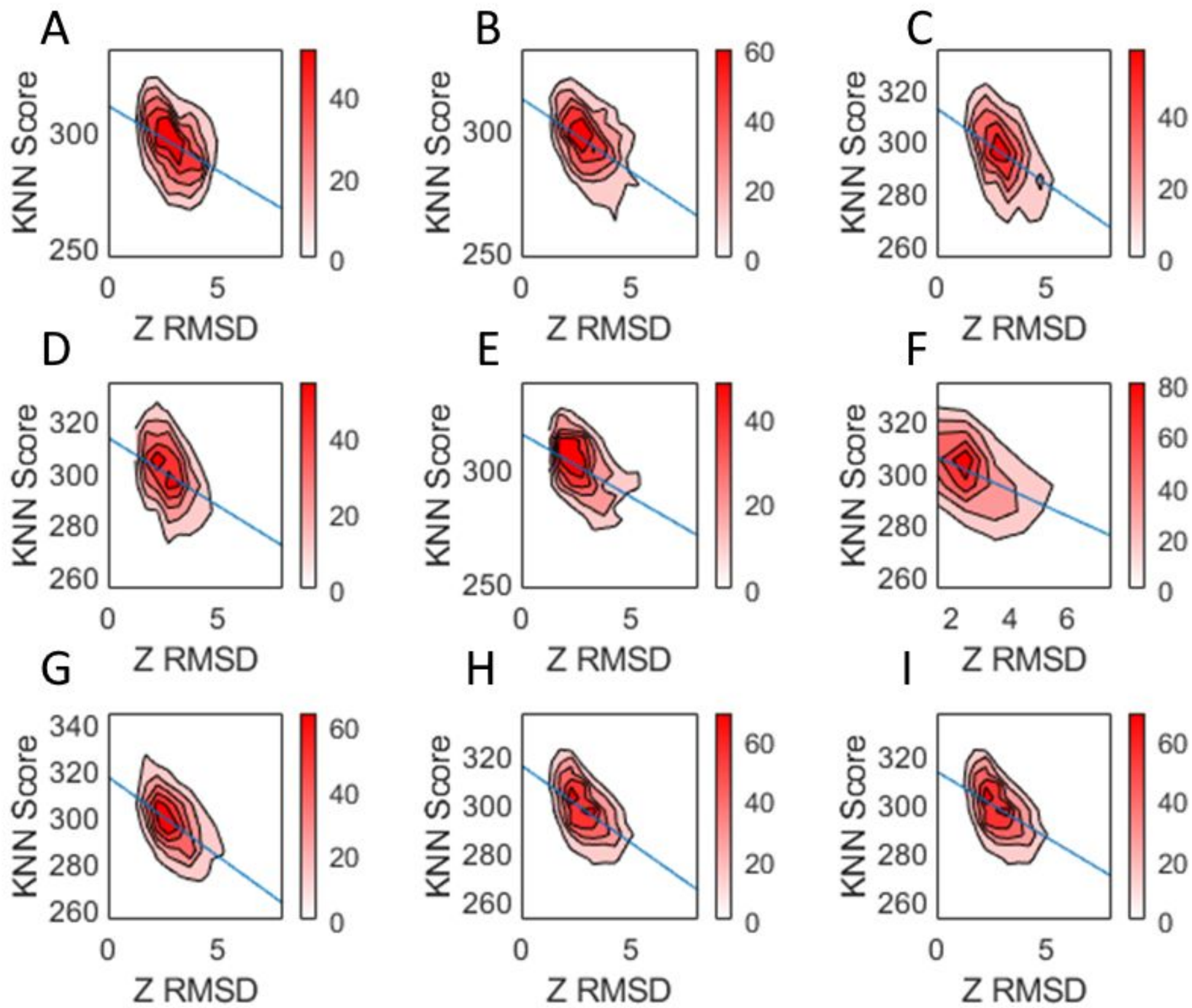

Figure S6) Subsets of the revPBE data set with SVP basis set and LYP correlation—the data from the three replicate bilayer snapshots. Panels A-C are the first snapshot, D-F are the second snapshot, and G-I are the third snapshot. A D and G are the upper leaflet (1:50 – 1:50), B E and H are the upper leaflet compared to the lower leaflet (1:50 – 51:100), and C F and I are the lower leaflet (51:100 – 51:100). The R^2 for the graphs are A is 0.15, B is 0.21, C is 0.24, D is 0.14, E is 0.19, F is 0.20, G is 0.28, H is 0.26, I is 0.17.

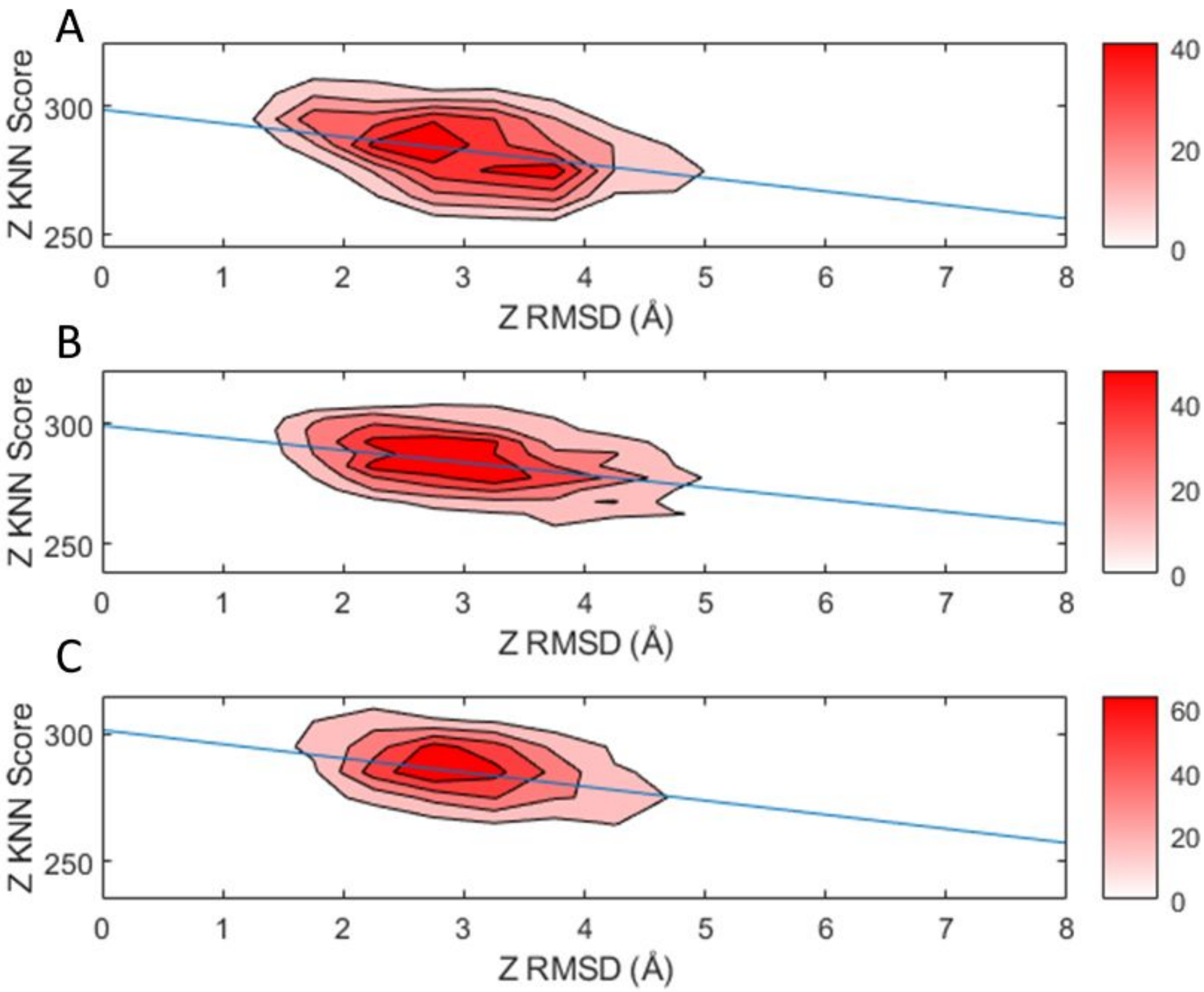

Figure S7) Subsets of the HF-3c data set with MINX basis set and D3 dispersion correction, geometric counterpoise correction, and short-range correction for deficiencies of small basis sets. Where panel A is the upper leaflet (1:50 – 1:50) with an R^2 of 0.12, panel B is the upper leaflet compared with the lower leaflet (1:50 – 51:100) with an R^2 of 0.13, and panel C is the lower leaflet compared with the lower leaflet (51:100 – 51:100) and has an R^2 of 0.14.

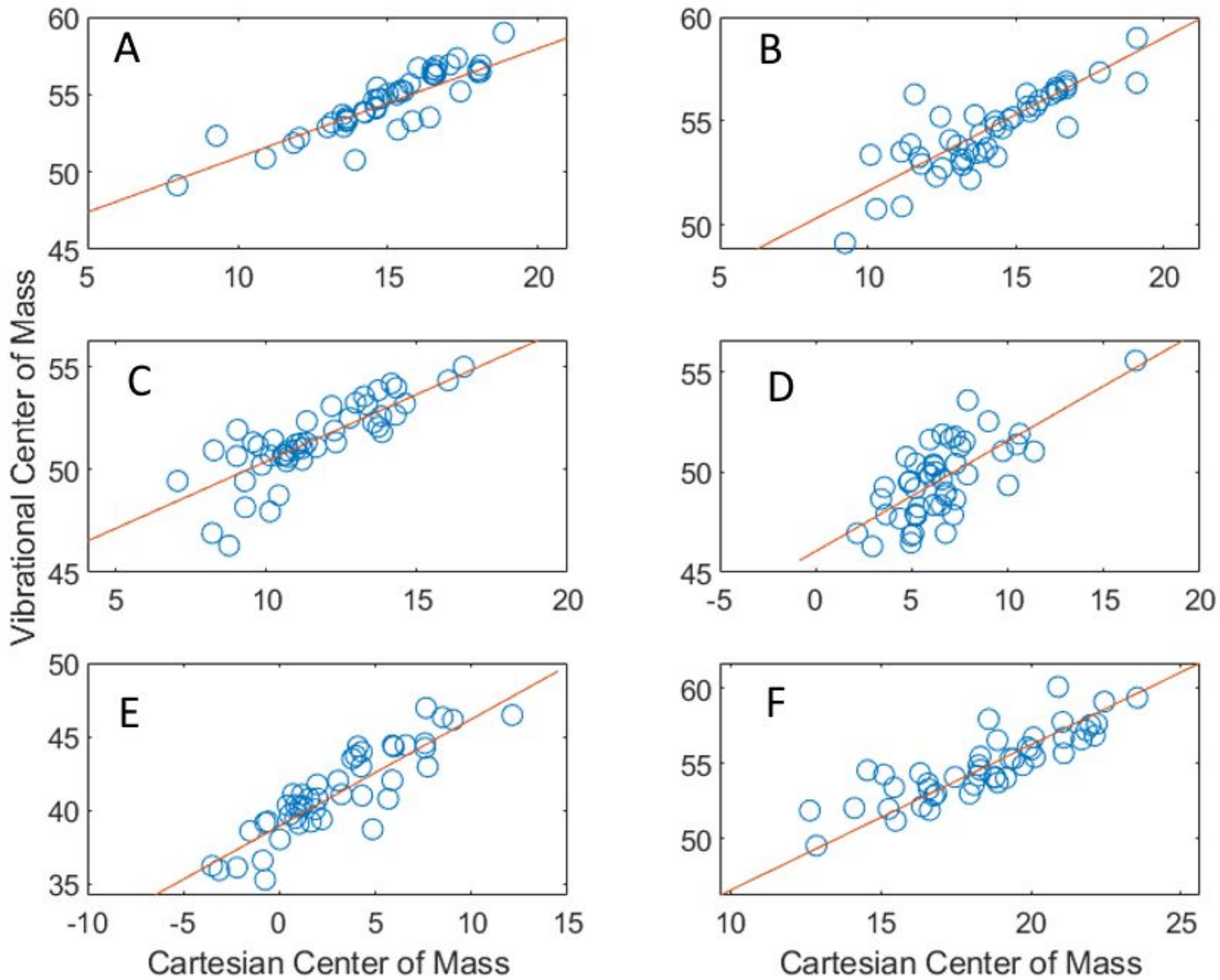

Figure S8) Different functional groups selected by their wavenumber and order-of-positions, including the positions of two carbonyls, all axis units in Angstroms. In the DFT simulations with revPBE and LYP correlation, the Sn1 carbonyl is at a higher frequency, around 1764 cm-1, and the Sn2 carbonyl is at a lower frequency, around 1716 cm-1. Panels from upper left to lower right: A) carbonyl 1 (Sn2) R^2=0.70, B) carbonyl 2 (Sn1) R^2=0.77, C) CH2 R^2=0.56, D) double bond R^2=0.49, E) methyl R^2=0.73, F) head group R^2=0.76. This figure shows the converged lipids from 1 to 50 in bilayer 1.

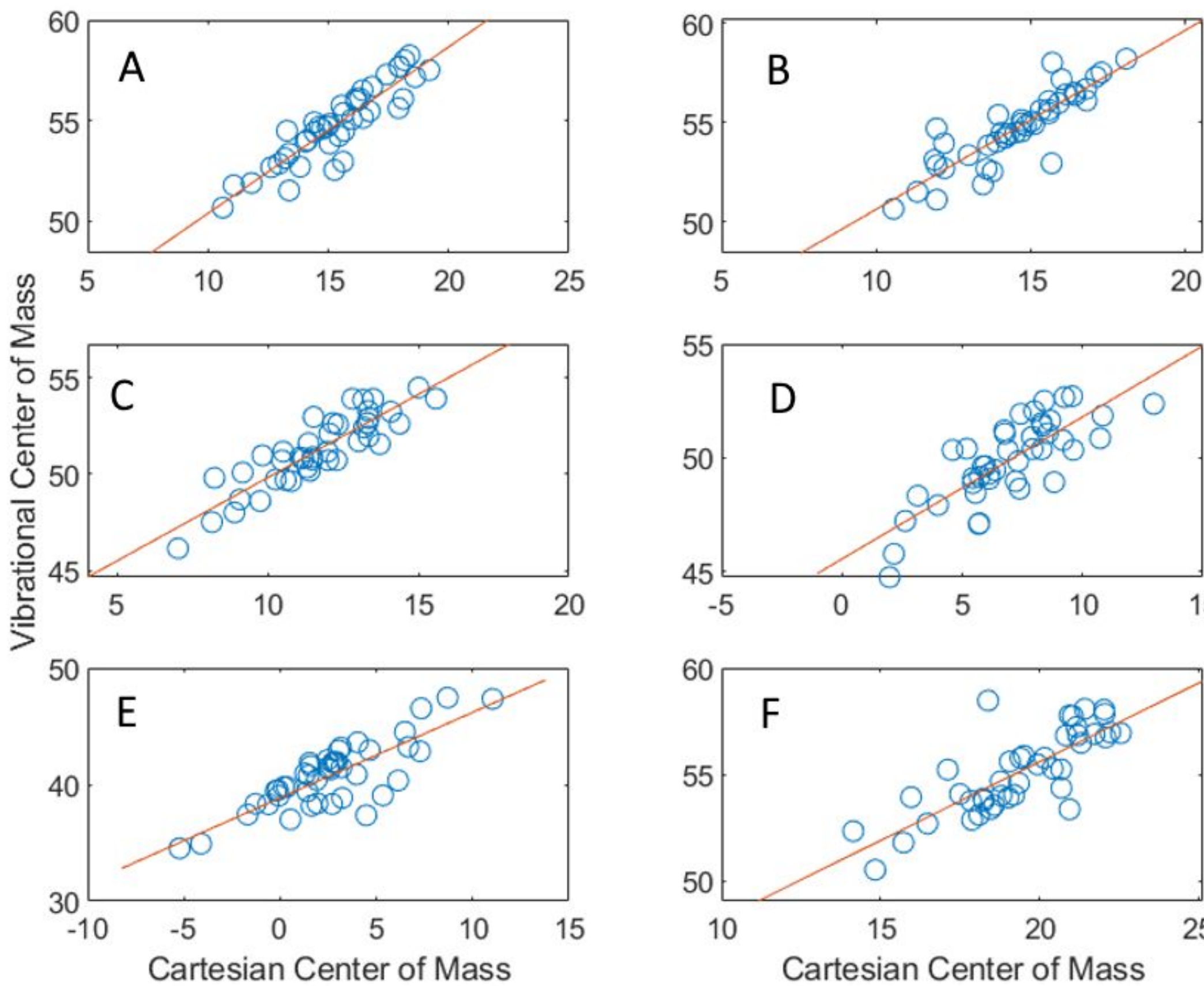

Figure S9) Different functional groups selected by their wavenumber and order-of-positions, including the positions of two carbonyls, all axis units in Angstroms. In the DFT theory revPBE with LYP correlation, the Sn1 carbonyl is at a higher frequency, 1764 cm^{-1}, and the Sn2 carbonyl is at a lower frequency, 1716 cm^{-1}. Panels from upper left to lower right: A) carbonyl 1 (Sn2) R^2=0.82, B) carbonyl 2 (Sn1) R^2=0.75, C) CH2 R^2=0.79, D) double bond R^2=0.63, E) methyl R^2=0.66, F) head group R^2=0.63. This figure shows the converged lipids from 1 to 50 in bilayer 2.

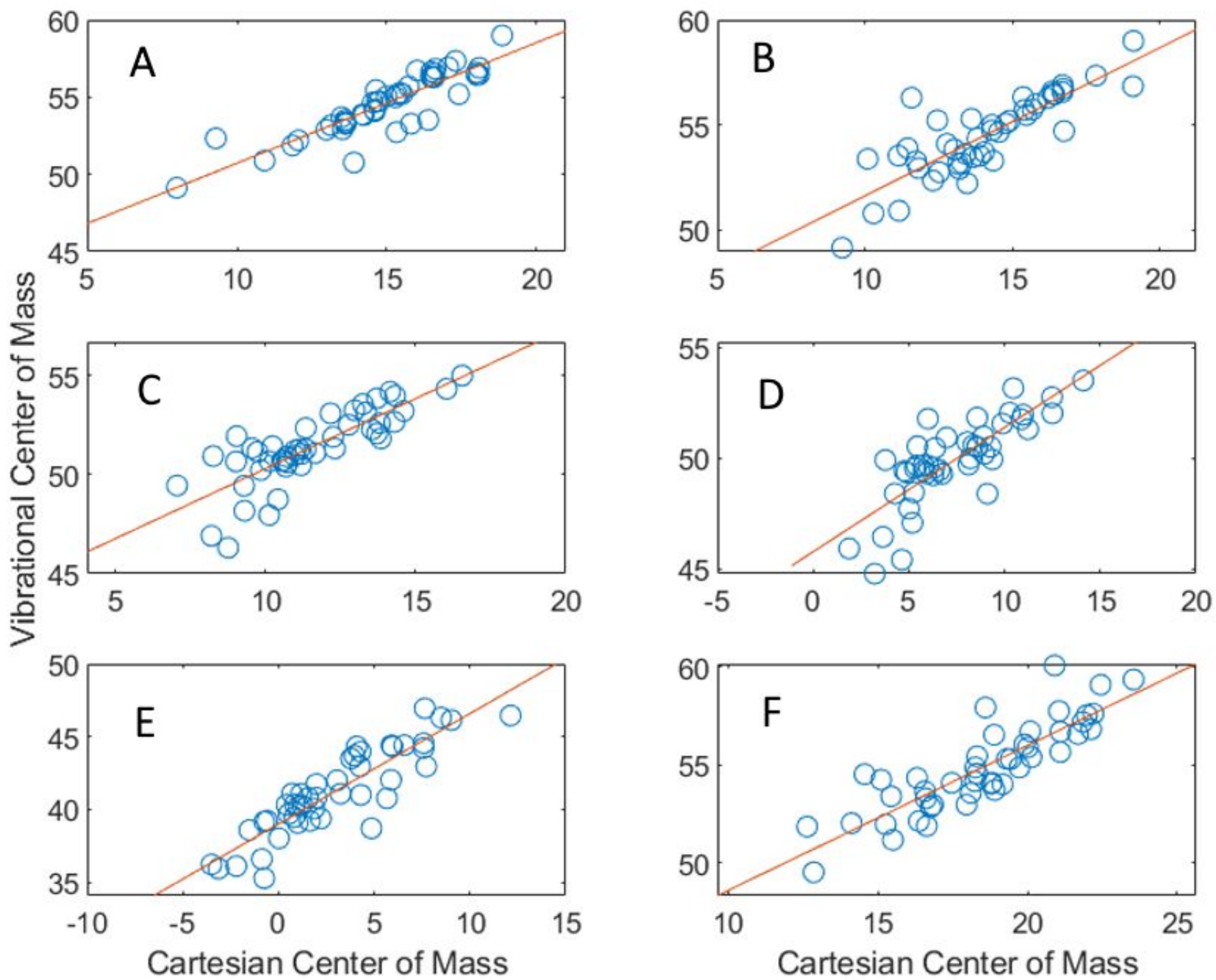

Figure S10) Different functional groups selected by their wavenumber and order-of-positions, including the positions of two carbonyls, all axis units in Angstroms. In the DFT theory revPBE with LYP correlation, the Sn1 carbonyl is at a higher frequency, 1764 cm^{-1}, and the Sn2 carbonyl is at a lower frequency, 1716 cm^{-1}, in the simulations. Panels from upper left to lower right: A) carbonyl 1 (Sn2) R^2=0.77, B) carbonyl 2 (Sn1) R^2=0.71, C) CH2 R^2=0.66, D) double bond R^2=0.64, E) methyl R^2=0.78, F) head group R^2=0.73. This figure shows the converged lipids from 1 to 50 in bilayer 3.
